# Resolvin D1 supports skeletal myofiber regeneration via actions on myeloid and muscle stem cells

**DOI:** 10.1172/jci.insight.137713

**Published:** 2020-09-17

**Authors:** James F. Markworth, Lemuel A. Brown, Eunice Lim, Carolyn Floyd, Jacqueline Larouche, Jesus A. Castor-Macias, Kristoffer B. Sugg, Dylan C. Sarver, Peter C.D. Macpherson, Carol Davis, Carlos A. Aguilar, Krishna Rao Maddipati, Susan V. Brooks

**Affiliations:** 1 Department of Molecular & Integrative Physiology,; 2 Department of Orthopaedic Surgery,; 3 Department of Biomedical Engineering, and; 4 Department of Surgery, University of Michigan Medical School, Ann Arbor, Michigan, USA.; 5 Department of Cellular & Molecular Physiology, Johns Hopkins University School of Medicine, Baltimore, Maryland, USA.; 6 Department of Pathology, Lipidomics Core Facility, Wayne State University, Detroit, Michigan, USA.

**Keywords:** Inflammation, Muscle Biology, Eicosanoids, Macrophages, Skeletal muscle

## Abstract

Specialized proresolving mediators (SPMs) actively limit inflammation and expedite its resolution by modulating leukocyte recruitment and function. Here we profiled intramuscular lipid mediators via liquid chromatography-tandem mass spectrometry–based metabolipidomics following myofiber injury and investigated the potential role of SPMs in skeletal muscle inflammation and repair. Both proinflammatory eicosanoids and SPMs increased following myofiber damage induced by either intramuscular injection of barium chloride or synergist ablation–induced functional muscle overload. Daily systemic administration of the SPM resolvin D1 (RvD1) as an immunoresolvent limited the degree and duration of inflammation, enhanced regenerating myofiber growth, and improved recovery of muscle strength. RvD1 suppressed inflammatory cytokine expression, enhanced polymorphonuclear cell clearance, modulated the local muscle stem cell response, and polarized intramuscular macrophages to a more proregenerative subset. RvD1 had minimal direct impact on in vitro myogenesis but directly suppressed myokine production and stimulated macrophage phagocytosis, showing that SPMs can modulate both infiltrating myeloid and resident muscle cell populations. These data reveal the efficacy of immunoresolvents as a novel alternative to classical antiinflammatory interventions in the management of muscle injuries to modulate inflammation while stimulating tissue repair.

## Abstract 2

Systemic administration of the immunoresolvent resolvin D1 enhances skeletal muscle repair via modulatory effects on both resident muscle stem cells and intramuscular macrophages.

## Introduction

Skeletal muscle damage induces an acute inflammatory response (1). Polymorphonuclear cells (PMNs) appear first within injured muscle, followed by blood monocyte–derived macrophages that invade within necrotic myofibers to engulf and clear tissue debris (2). In addition to preparing the injury site to enable subsequent repair, phagocytosis triggers a switch to a reparative macrophage subset that supports muscle regeneration via interactions with postmitotic muscle cells (myofibers) and associated resident myogenic muscle stem cells (MuSCs) (3). Unlike macrophages, PMNs have overall deleterious effects on muscle regeneration (4). Therefore, clearance of PMNs from regenerating muscle is important for the inflammatory response to be successfully resolved.

Resolution of the inflammatory response has been classically considered a largely passive event (5). More recently, however, demonstration of the production of distinct families of specialized proresolving lipid mediators (SPMs) during the resolution phase of the inflammatory response suggests that it is actively regulated (6). SPMs are a superfamily of bioactive metabolites of essential fatty acids, including the lipoxins, resolvins, protectins, and maresins (7). These autocoids actively limit PMN influx, while simultaneously stimulating macrophage functions essential for timely resolution of inflammation (8). Emerging evidence suggests that SPMs can also exert important biological actions on nonimmune cell populations (e.g., stem cells) that may contribute to their physiological effects (9).

The role of lipid mediators in skeletal muscle inflammation and repair is poorly understood (10). Prior studies have largely focused on the prostaglandins, proinflammatory eicosanoids synthesized via the cyclooxygenase-1 and -2 (COX-1 and COX-2) pathways (11). Given the importance of inflammation in the control of muscle repair, it is not surprising that treatment with NSAIDs can impede muscle regeneration (12). The discovery of SPMs provides the potential for novel strategies to modulate inflammation that differ fundamentally from this antiinflammatory approach (13). Administration of resolution agonists, coined immunoresolvents, can limit inflammation and expedite its resolution, while relieving pain (14). In contrast to the inhibitory actions of NSAIDs on muscle repair, SPMs have proregenerative actions in a number of tissues (15).

SPMs are produced in humans in response to physiological stress (e.g., exercise) (16, 17) as well as following sterile muscle injury in mice (18). In addition, Giannakis et al. 2019 reported that, in mice with a regeneration defect due to a lack of MuSCs, intramuscular injection of one native SPM, resolvin D2 (RvD2), accelerated macrophage transitions and improved recovery of muscle mass and strength following muscle injury (19). However, the therapeutic applicability of immunoresolvents to modulate muscle inflammation and repair under physiological circumstances in which MuSCs are indispensable for muscle repair remains unknown (20). Moreover, the Giannakis study (19) focused on immunological endpoints; thus there remains a complete lack of data concerning the impact of immunoresolvents on cellular and molecular indices of myofiber regeneration or the MuSC response.

The goal of the present study, therefore, was to investigate the potential role of SPMs in adaptive myofiber hypertrophy and myofiber degeneration/regeneration. Based on recent reports that the proresolving peptide annexin A1, which shares its cell surface receptor (FPR2/ALX) with the SPM resolvin D1 (RvD1), positively regulates skeletal muscle regeneration (21, 22), we also tested whether daily systemic administration in healthy young mice of RvD1, as an immunoresolvent, modulates cellular and molecular indices of myofiber regeneration and myogenic MuSC responses to muscle injury in vivo and whether RvD1 directly modulates myogenesis and myokine production in vitro.

## Results

### Temporal changes in muscle lipid mediators following injury.

**Figure 1:**
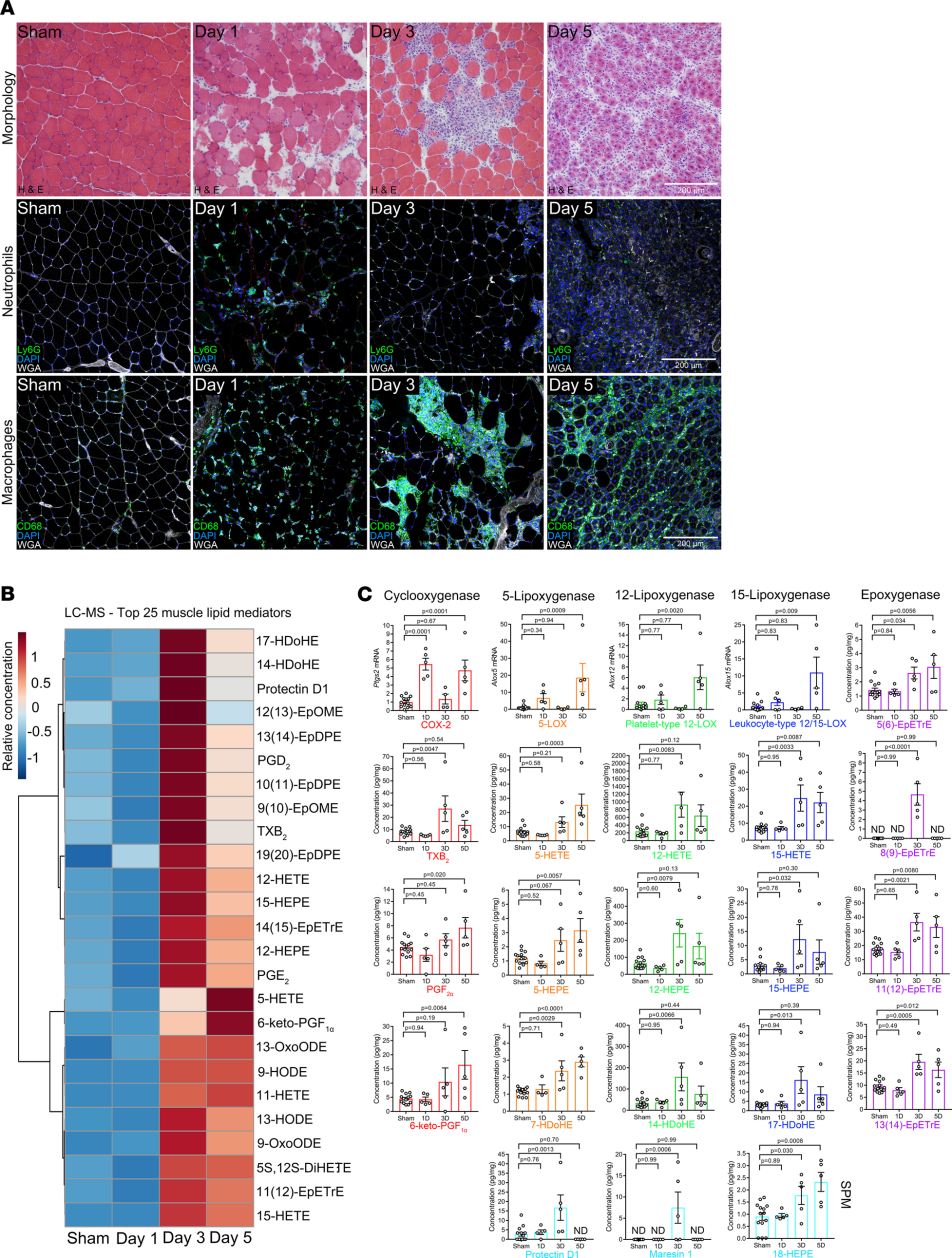
Dynamic shifts in muscle lipid mediators following skeletal muscle injury. (**A**) C57BL/6 mice received bilateral intramuscular injection of the tibialis anterior (TA) muscle with 50 μL of 1.2% barium chloride (BaCl_2_) to induce myofiber injury. Control mice received bilateral sham intramuscular injections with 50 μL of sterile saline. TA cross sections were stained for H&E, polymorphonuclear cells (PMNs, Ly6G), or monocytes/macrophages (CD68). Scale bars: 200 μm. (**B**) Heatmap of the top 25 muscle lipid mediators (based on partial least squares discriminant statistical analysis variable importance projection [PLS-DA VIP] score) modulated by muscle injury as determined by liquid chromatography-tandem mass spectrometry (LC-MS/MS). (**C**) Muscle mRNA expression of major lipid mediator biosynthetic enzymes and respective downstream lipid metabolites including cyclooxygenase-2 (COX-2, red), 5-lipoxygenase (5-LOX, orange), platelet-type 12-lipoxygenase (12-LOX, green), leukocyte-type 12/15-lipoxygenase (12/15-LOX, dark blue), and epoxygenase (CYP 450, purple) pathways. Downstream bioactive specialized proresolving lipid mediators (SPMs) detected in muscle are also shown (light blue). Bars show the mean ± SEM of 4–5 mice per group with dots representing data from each mouse. The sham injury time course was pooled for quantitative analysis. *P* values are by 1-way ANOVA followed by Holm-Šidák post hoc tests with sham-injured mice serving as controls. ND, below the limits of detection.

Intramuscular barium chloride (BaCl_2_) injection resulted in widespread myofiber damage and local appearance of both PMNs (Ly6G^+^ cells) and macrophages (CD68^+^ cells) (Figure 1A). By day 3, there was progressive PMN clearance and robust macrophage infiltration, and many small, regenerating myofibers with characteristic centrally located nuclei appeared by day 5. COX-2 expression increased markedly at day 1 postinjury, but the overall intramuscular lipid mediator profile did not yet differ substantially from sham-injected control mice (Figure 1, B and C). By day 3, there were increased proinflammatory eicosanoids derived from the COX (e.g., thromboxane B_2_, prostaglandin E_2_ [PGE_2_], and prostaglandin D_2_) and 12-LOX pathways (e.g., 12-HETE). Muscle mRNA expression of 5-LOX, platelet-type 12-lipoxygenase (12-LOX), and leukocyte-type 12/15-LOX increased at later points following injury. This resulted in higher concentrations in injured compared with control muscles of LOX-generated monohydroxylated pathway intermediates in the biosynthesis of proinflammatory leukotrienes (5-HETE), as well as SPMs, including the lipoxins (15-HETE), E-series resolvins (18-HEPE), D-series resolvins/protectins (17-HDoHE), and maresins (14-HDoHE) (Figure 1C). Downstream bioactive SPMs, including protectin D1 and maresin 1, were also detected at day 3 postinjury, while lipoxins, E-resolvins, and D-resolvins were generally below the limits of detection within muscle (Supplemental Table 1; supplemental material available online with this article; https://doi.org/10.1172/JCI13771DS1). In addition, we detected for the first time to our knowledge elevated epoxygenase (CYP p450) metabolites in injured muscle (Figure 1C and Supplemental Table 1).

### Dynamic changes in muscle lipid mediators are conserved across different species and models of injury.

**Figure 2:**
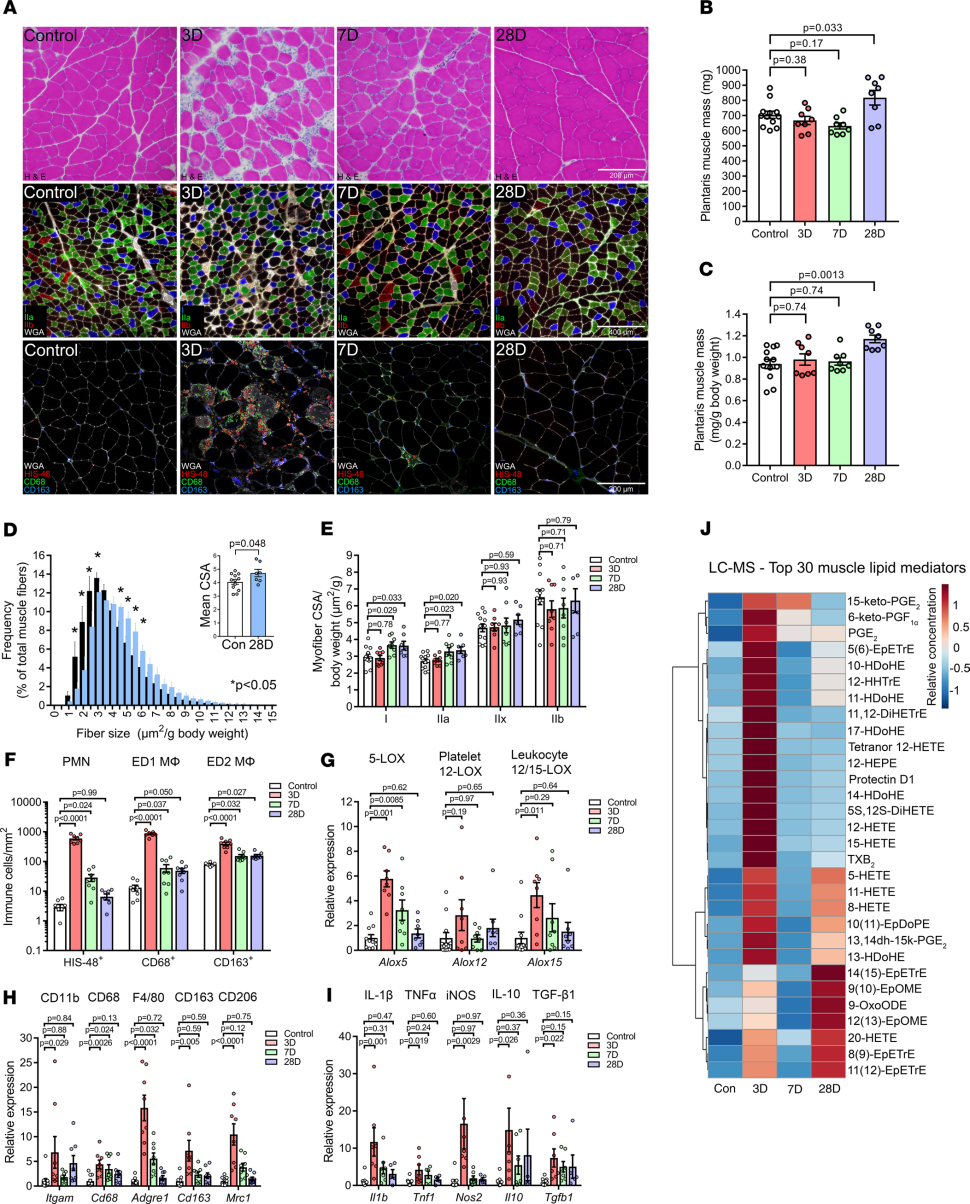
Muscle lipid mediator responses to functional plantaris overload. (**A**) Sprague-Dawley rats underwent bilateral functional overload of the plantaris muscle via synergist ablation surgery. Plantaris muscles from age- and sex-matched rats served as nonsurgical controls. Muscle cross sections were stained for H&E, muscle fiber type, or inflammatory cells, including PMNs (HIS48), ED1 monocyte/macrophages (CD68), and ED2 macrophages (CD163). Type IIx fibers remain unstained (black). Scale bars: 200 μm (top, bottom), 400 μm (middle). (**B** and **C**) Absolute and relative plantaris muscle mass following functional overload. (**D**) Frequency distribution of cross sectional area (CSA) of total muscle fiber population in plantaris muscles of control and day 28 post–synergist ablation rats. Inset shows the mean myofiber CSA. (**E**) Mean myofiber CSA of split by respective muscle fiber type. (**F**) Quantification of intramuscular PMNs (HIS48^+^ cells), inflammatory ED1 macrophages (CD68^+^ cells), and resident/M2-like ED2 macrophages (CD163^+^ cells). (**G**) Whole-muscle mRNA expression of 5-LOX, 12-LOX, and 12/15-LOX. (**H** and **I**) Muscle mRNA expression of immune cell markers, cytokines, and markers of macrophage activation state. Gene expression was normalized to *Gapdh*. (**J**) Heatmap of the top 30 muscle lipid mediators (based on PLS-DA VIP score) modulated by functional overload as determined by LC-MS/MS analysis. Bars show the mean ± SEM of 8–12 plantaris muscles from 4–6 rats per group. Dots represent data from each muscle. *P* values were determined 1-way ANOVA followed by Holm-Šidák post hoc tests with nonsurgery rats serving as a control group (**B**, **C**, and **E**–**I**) or by 2-tailed unpaired *t* tests (**D**).

We also performed lipidomic profiling of the plantaris muscle following synergist ablation–induced functional overload in rats. This is a milder, but potentially more physiologically relevant, model of myofiber damage when compared with BaCl_2_-induced injury (Figure 2A). Synergist ablation resulted in an increase in the mass of the overloaded plantaris at 28 days postsurgery (Figure 2, B and C), due to increased myofiber size (Figure 2D), which was most evident for type I and IIa fiber types (Figure 2E). Control plantaris muscles contained many resident ED2 (CD163^+^) macrophages, few scattered ED1 (CD68^+^) macrophages, and very few PMNs (HIS48^+^ cells). Therefore, unlike in mice, the resident macrophages in rat muscle were predominantly CD68^–^CD163^+^ rather than CD68^+^CD163^+^ cells (Supplemental Figure 1). Three days postsurgery, overloaded muscles showed localized inflammation (Figure 2A and Supplemental Figure 2A), with at least 3 distinct myeloid cell populations present, including PMNs (HIS48^+^ cells), ED1 macrophages (CD68^+^CD163^–^ cells), and ED2 macrophages (CD68^–^CD163^+^ cells) (Figure 2F). Scattered HIS48^+^ cells could still be seen at day 7 but were absent by day 28. CD68^+^ and CD163^+^ cells persisted, albeit in much lower numbers, at both 7 and 28 days of recovery, with a clear increase in coexpression of CD68 and CD163 by the remaining macrophages (Supplemental Figure 2B).

Plantaris overload increased mRNA expression of major 5-, 12-, and 15-LOX enzymes (Figure 2G), immune cell markers (Figure 2H), and cytokines (Figure 2I). Lipidomic profiling also showed elevated intramuscular lipid mediators from the COX, LOX, and CYP pathways, including many proinflammatory eicosanoids (e.g., PGE_2_) as well as pathway markers of SPM biosynthesis including lipoxins (15-HETE), D-series resolvins/protectins (17-HDoHE), and maresins (14-HDoHE) (Figure 2J and Supplemental Table 1). Lipoxin A_4_, protectin D1, maresin 1, resolvin D6, and 8-oxo-RvD1 were also detected (Supplemental Table 1). While most COX and LOX metabolites had returned to resting levels by day 7, many CYP pathway metabolites remained elevated at day 28 (Figure 2J and Supplemental Table 1).

### Systemic resolvin D1 treatment limits muscle inflammation.

**Figure 3:**
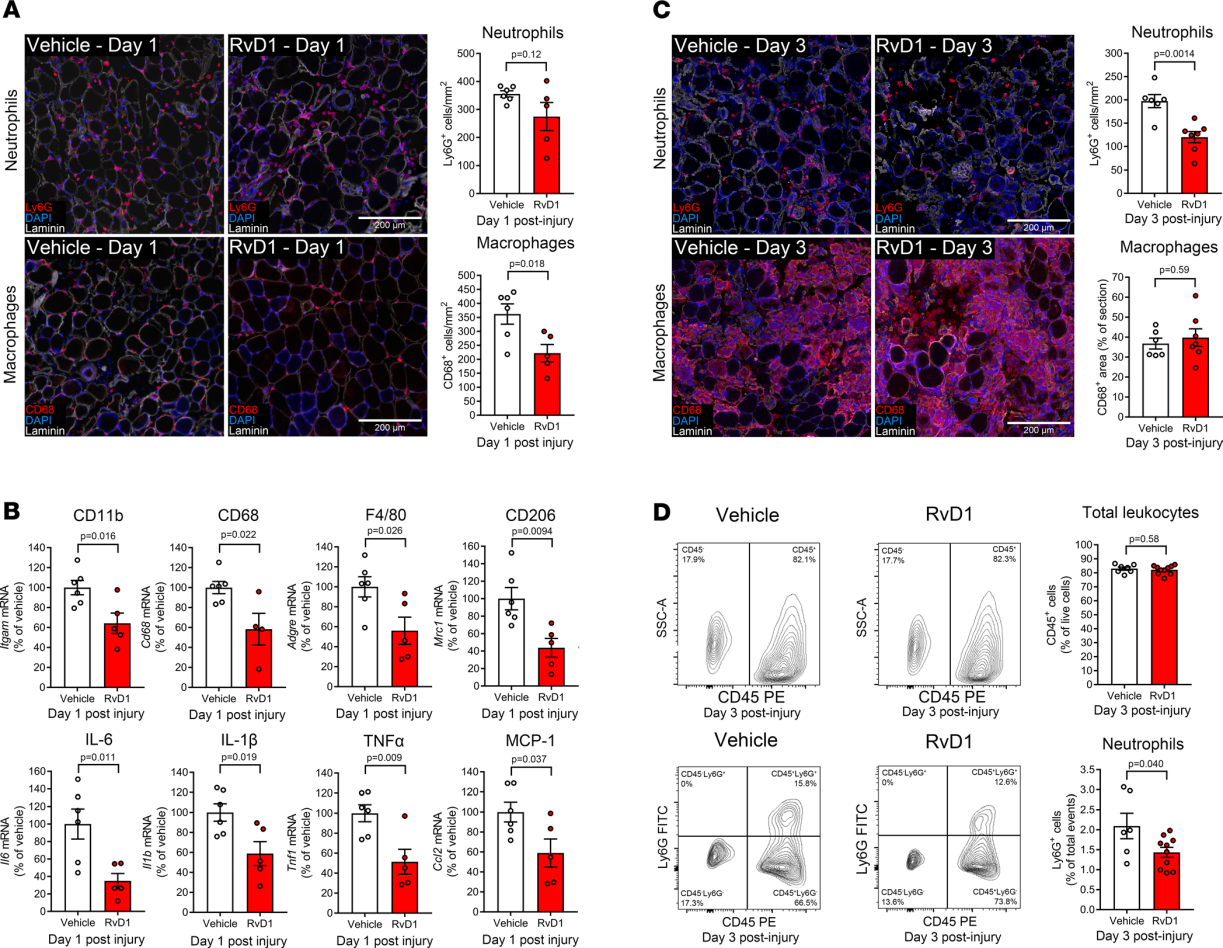
Resolvin D1 limits inflammation and expedites its resolution following muscle injury. (**A**) C57BL/6 mice received bilateral intramuscular injection of the TA muscle with 50 μL of 1.2% BaCl_2_ to induce myofiber injury. Mice were treated with RvD1 (100 ng) or vehicle (0.1% ethanol) via intraperitoneal (IP) injection approximately 5 minutes before muscle injury. TA cross sections were stained for PMNs (Ly6G) or monocytes/macrophages (CD68). Cell nuclei and the basal lamina were counterstained with DAPI and laminin antibody, respectively. Scale bars: 200 μm. (**B**) Whole-muscle mRNA expression of immune cell markers and inflammatory cytokines at 24 hours postinjury as determined by real-time quantitative reverse transcription PCR (RT-qPCR). Gene expression was normalized to *Actb*. (**C**) Mice were treated for 72 hours with daily IP injection of RvD1 (100 ng) or vehicle (0.1% ethanol) following muscle injury, and TA cross sections were stained for PMNs (Ly6G) or macrophages (CD68) at day 3 postinjury. Representative images show examples of regions of muscle not yet infiltrated by macrophages where lingering PMNs remained (top) and macrophage-rich regions largely devoid of PMNs (bottom). (**D**) Single cells isolated from pooled left and right TA muscles at day 3 postinjury were analyzed by flow cytometry for CD45-PE and Ly6G-FITC. Bars show the mean ± SEM of 5–10 mice per group with dots representing data from each mouse. *P* values were determined 2-tailed unpaired *t* tests.

We next investigated the ability of SPMs to alter muscle inflammation using the BaCl_2_ injury model in which muscle inflammation was uniform and widespread. Mice were treated with RvD1, an SPM derived from n-3 docosahexaenoic acid (DHA) (23), via the sequential actions of the 15- and 5-LOX pathways (24). We chose RvD1 because of its established dose-response pharmacokinetics in vivo (25), extensively documented receptor-dependent proresolving actions (26), and well-documented therapeutic efficacy with systemic administration in mice (27). Intraperitoneal (IP) injection of RvD1 at the time of BaCl_2_ injury blunted accumulation of intramuscular macrophages (CD68^+^ cells) 24 hours later but did not affect PMN (Ly6G^+^ cell) number (Figure 3A). RvD1 treatment also reduced muscle mRNA expression of immune cell markers induced by BaCl_2_, including CD11b, CD68, F4/80, and CD206 (Figure 3B), as well as cytokines, such as IL-6, IL-1β, monocyte chemoattractant protein 1 (MCP-1), and TNF-α (Figure 3B). Common housekeeping genes, including β-actin and 18S, were markedly induced in response to BaCl_2_ injection, and RvD1 treatment reduced 18S but not β-actin expression (data not shown). Therefore, RT-qPCR data in Figure 3 were normalized to *Actb* and presented relative to injured vehicle-treated mice.

### Resolvin D1 expedites clearance of intramuscular PMNs.

To investigate the effect of RvD1 on the resolution phase of the inflammatory response, we treated mice daily with IP injections of RvD1 for 72 hours after BaCl_2_-induced muscle injury. RvD1-treated mice showed approximately 40% fewer intramuscular PMNs, while the relative area of the muscle cross section containing CD68^+^ staining was similar between RvD1- and vehicle-treated groups (Figure 3C). Intramuscular single cells from TA muscles collected at day 3 postinjury were also analyzed by flow cytometry (Figure 3D). Approximately 80% of the live single cells within muscle stained positive for the panleukocyte marker CD45, and RvD1 treatment did not alter total leukocytes. Nevertheless, the proportion of CD45^+^ cells that expressed Ly6G was lower in RvD1-treated mice, and this was accompanied by a parallel increase in the relative proportion of intramuscular CD45^+^Ly6G^–^ cells (Figure 3D).

### Resolvin D1 stimulates macrophage phagocytosis.

**Figure 4:**
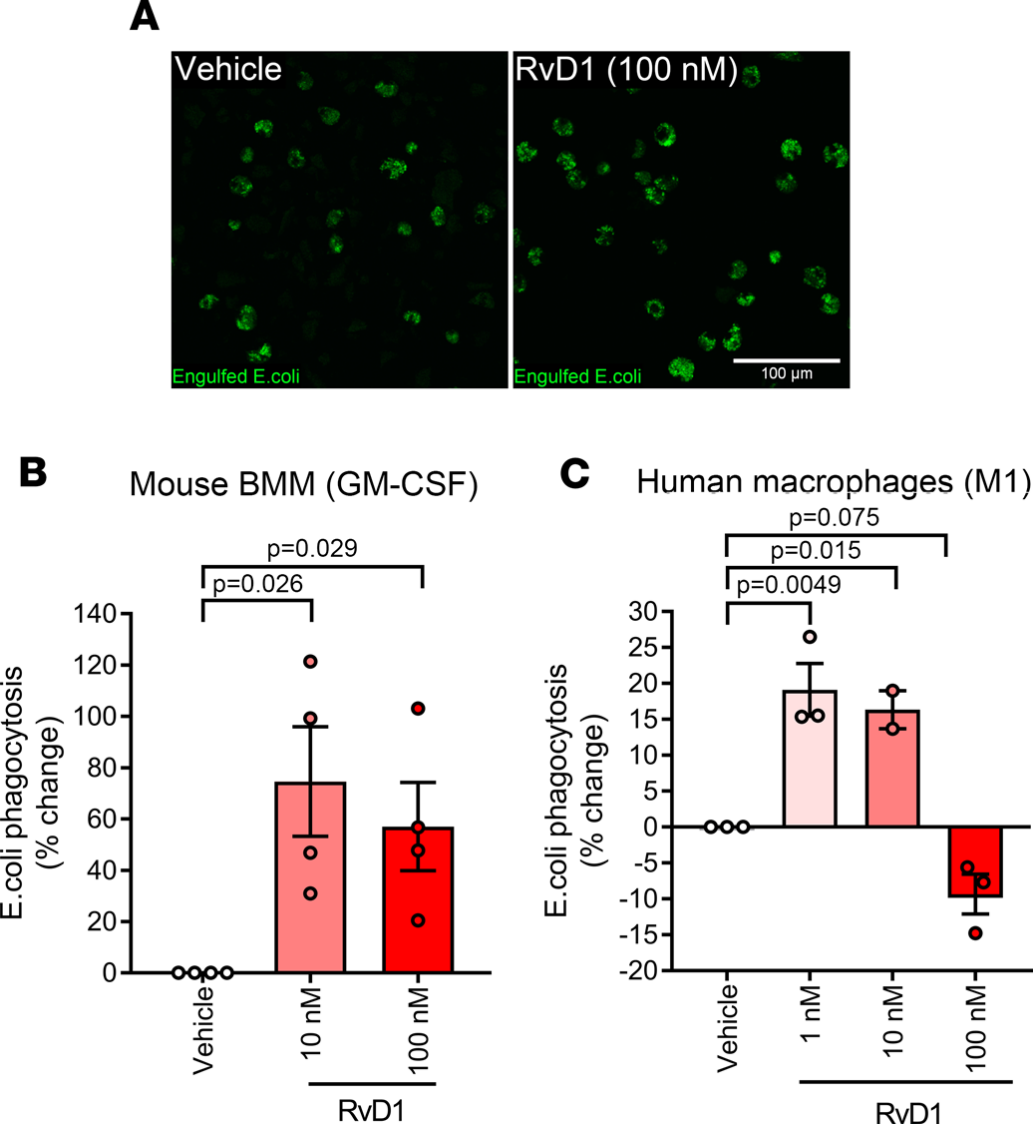
Resolvin D1 enhances macrophage phagocytosis. (**A**) Bone marrow–derived macrophages (BMMs) from C57BL/6 mice were obtained by culturing myeloid precursors for 7 days in the presence of 20 ng/mL GM-CSF. BMMs were then pretreated for 15 minutes with RvD1 (100 nM) before incubation with pHrodo Green *E*. *Coli* Bio Particles for 60 minutes at 37°C in the continued presence of RvD1. (**B**) Quantification of phagocytosis by mouse GM-CSF BMMs treated with RvD1 (10–100 nM) as determined using a 96-well fluorescent plate reader (excitation/emission of 509/533 nm). (**C**) The effect of increasing doses of RvD1 (1–100 nM) on phagocytosis by human PBMC-derived M1-polarized macrophages. Data are presented as the percentage change in fluorescence intensity relative to matching vehicle-treated macrophage cultures from the same host. Bars show the mean ± SEM of cells from 3–4 donors with dots with representing data from each host. *P* values were determined by 1-way ANOVA followed by Holm-Šidák post hoc tests with vehicle-treated cells serving as a control group.

Macrophages play an important role in the active resolution of inflammation via their phagocytic uptake and removal of pathogens, cellular debris, and apoptotic PMNs. Therefore, we assessed whether RvD1 could directly influence macrophage phagocytosis in vitro (Figure 4A). RvD1 at doses between 10 and 100 nM (based on prior studies, refs. 26, 28) stimulated phagocytosis by GM-CSF–derived mouse BMMs (Figure 4B). RvD1 (1–10 nM) also stimulated phagocytosis by M1-polarized macrophages derived from human PBMCs, although higher doses of RvD1 (100 nM) were apparently refractory in this cell type (Figure 4C).

### Expedited inflammation-resolution supports regenerating myofiber growth.

**Figure 5:**
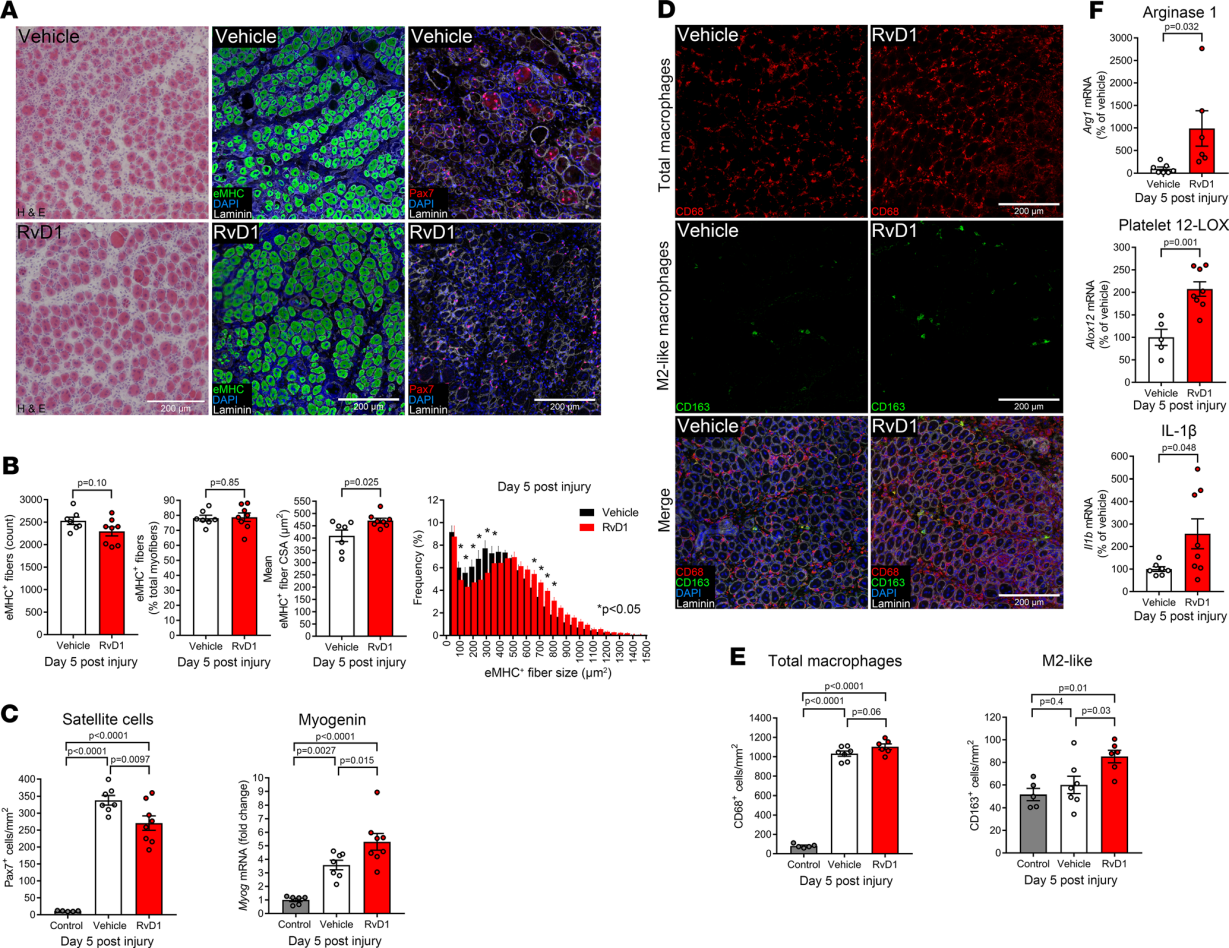
Resolvin D1 enhances myofiber regeneration by modulating muscle stem cells and macrophages. (**A**) C57BL/6 mice received bilateral intramuscular injection of the TA muscle with 50 μL of 1.2% BaCl_2_ to induce myofiber injury. Mice were treated with RvD1 (100 ng) or vehicle (0.1% ethanol) by IP injection for 5 days. TA cross sections were stained for H&E, eMHC, or Pax7 to stain satellite cells (MuSCs). Cell nuclei and the basal lamina were counterstained with DAPI and laminin antibody, respectively. Scale bars: 200 μm. (**B**) Quantitative analysis of total regenerating (eMHC^+^) myofiber number, relative eMHC^+^ fiber number (as % of total fibers), mean eMHC^+^ fiber CSA, and the percentage frequency distribution of the CSA of the eMHC^+^ fiber population. (**C**) Quantification of MuSC number (Pax7^+^DAPI^+^ nuclei) and muscle mRNA expression of the myogenic regulatory factor myogenin at day 5 postinjury. (**D**) Cross sections of TA muscles at day 5 postinjury were stained for total macrophages (CD68) and M2-like macrophages (CD163). (**E**) Quantification of total intramuscular macrophages (CD68^+^ cells) and M2-like macrophages (CD163^+^ cells). (**F**) Muscle mRNA expression of macrophage-related genes including arginase-1 (Arg1), 12-LOX, and IL-1β. Gene expression was normalized to *Actb*. Bars show the mean ± SEM of 5–8 mice per group with dots representing data for each mouse. *P* values were determined by 1-way ANOVA followed by pairwise Holm-Šidák post hoc tests (**C** and **E**) or by 2-tailed unpaired *t* tests (**B** and **F**).

To test whether stimulating resolution of inflammation influenced myofiber regeneration, mice were treated daily with daily IP administration of RvD1, and TA muscles were collected 5 days following injection with BaCl_2_ or vehicle. H&E staining of TA muscle cross sections revealed no gross histological differences between vehicle- and RvD1-treated mice (Figure 5A). Thus, RvD1 did not appear to obviously perturb normal muscle regeneration as has been reported with systemic NSAID treatment (29). To quantify the extent of myofiber regeneration, tissue sections were stained for embryonic myosin heavy chain (eMHC) (Figure 5A and Supplemental Figure 3). RvD1 did not influence the number of eMHC^+^ fibers but did increase the average CSA of the regenerating (eMHC^+^) myofibers (Figure 5B).

### Immunoresolvent treatment modulates muscle stem cell responses to injury.

Skeletal muscle regeneration is dependent on the function of resident MuSCs (20). Therefore, we also stained muscle cross sections with an antibody against the MuSC marker Pax7 (Figure 5A). The number of Pax7^+^ cells increased approximately 20-fold at day 5 following BaCl_2_ injury and RvD1 treatment reduced MuSC number (Figure 5C). Because myogenic regulatory factors control the activation, proliferation, and differentiation of MuSCs, we questioned whether RvD1 also influenced myogenic gene expression. Whole-muscle mRNA expression of myogenin increased markedly at day 5 postinjury and increased even further in mice that received RvD1 (Figure 5C). Taken together with the effect of RvD1 to increase regenerating myofiber size, a lower muscle Pax7^+^ cell density at day 5 postinjury may be interpreted as RvD1 stimulating differentiation and/or fusion of MuSCs with regenerating myofibers.

### Resolvin D1 shifts intramuscular macrophage activation state.

Many macrophages persisted in muscle at day 5 after BaCl_2_ injury, and RvD1 did not influence total muscle macrophages (Figure 5E). Only a small proportion of intramuscular macrophages coexpressed CD163 (≤5%) (Figure 5D). Nevertheless, RvD1-treated mice had greater numbers compared with vehicle-treated mice of CD163^+^ macrophages (Figure 5E). RvD1 also increased muscle mRNA expression of the M2 macrophage marker arginase-1 (Arg-1) and 12-LOX, a key SPM biosynthetic enzyme that is highly expressed by mature macrophages (Figure 5F). Expression of the macrophage-related proinflammatory cytokine IL-1β was also ~3-fold higher in regenerating muscle of RvD1-treated mice (Figure 5F).

### Improved recovery of muscle function in resolvin D1–treated mice.

**Figure 6:**
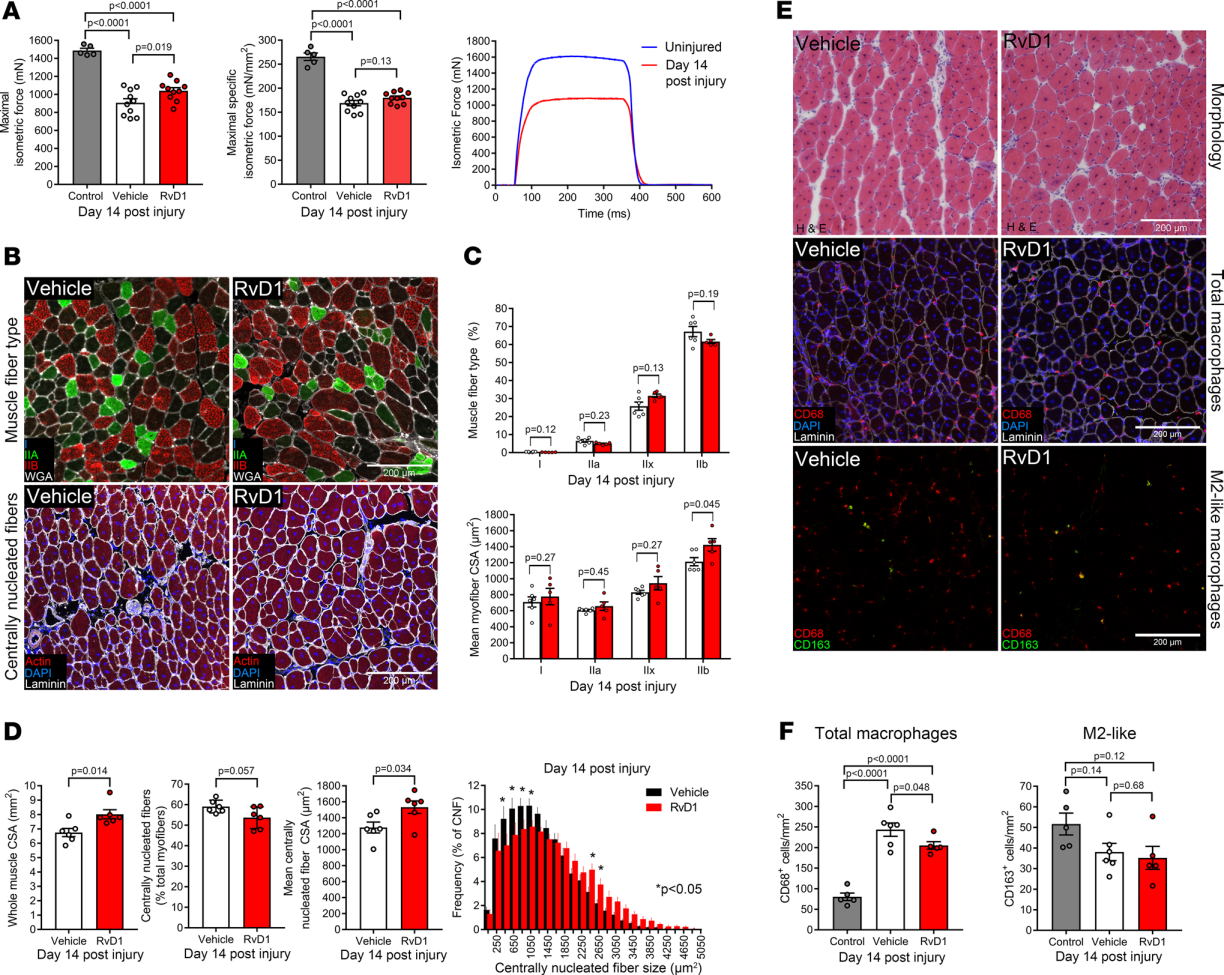
Resolvin D1 improves recovery of isometric muscle strength. (**A**) C57BL/6 mice received bilateral intramuscular injection of the TA muscle with 50 μL of 1.2% BaCl_2_ to induce myofiber injury. Mice were treated with RvD1 (100 ng) or vehicle (0.1% ethanol) for 14 days. TA muscle function was tested for maximal isometric nerve-stimulated in situ contractile force (P_o_) with age- and sex-matched mice serving as uninjured controls. Absolute maximal isometric force (mN) generated by the TA muscle was measured and used to calculate maximal specific isometric contractile force (sP_o_, mN/mm^2^). Representative force traces obtained from uninjured and injured TA muscles are shown. (**B**) TA cross sections were stained with conjugated phalloidin to label the total muscle fiber population or for muscle fiber type with antibodies against MHC I, IIa, and IIb. Type IIx fibers remain unstained (black) and are identified by lack of fluorescence staining. Cell nuclei and the basal lamina were counterstained with DAPI and laminin antibody, respectively. Scale bars: 200 μm. (**C**) Quantitative analysis of percent muscle fiber type composition and mean fiber CSA split by muscle fiber type as determined by MuscleJ. (**D**) Quantification of overall TA muscle CSA, the percentage of centrally nucleated (regenerating) myofibers, mean regenerating myofiber CSA, and percent frequency distribution of regenerating fiber CSA as determined by MuscleJ. (**E**) TA cross sections were stained for H&E, total macrophages (CD68^+^ cells), and M2-like macrophages (CD163^+^ cells). Scale bars: 200 μm. (**F**) Quantification of the histological presence of total muscle macrophages and M2-like macrophages. Cell counts were performed manually throughout the entire muscle cross section and then normalized to tissue surface area as determined by MuscleJ. Bars show the mean ± SEM of 5–10 mice per group with dots representing data from each mouse. *P* values were determined by 1-way ANOVA followed by pairwise Holm-Šidák post hoc tests (**A** and **F**) or 2-tailed unpaired *t* tests (**C** and **D**).

BaCl_2_-induced muscle injury reduced absolute muscle force (P_o_) by approximately 40% at day 14 postinjury, and this force deficit persisted when normalized to muscle size (specific force, sP_o_) (Figure 6A). Treatment with RvD1 improved recovery of P_o_ by approximately 15% but did not influence recovery of sP_o_. To assess the cellular basis for this improvement in P_o_, TA cross sections at day 14 postinjury were analyzed by immunohistochemistry to determine regenerating muscle fiber type and myofiber CSA (Figure 6B). RvD1 did not alter the fiber type composition of regenerating muscle but did specifically increase the size of fast-twitch type IIb fibers (Figure 6C). There was also an improved recovery of overall TA muscle CSA in mice receiving RvD1 (Figure 6D). Approximately 60% of the muscle fibers contained centrally located myonuclei at this point; RvD1 did not influence the absolute or relative number of these regenerating myofibers, but CSA of regenerating myofibers was larger in mice treated with RvD1 (Figure 6D). Regenerating muscles still contained ~3-fold more macrophages than uninjured muscles (Figure 6E), and only a minority (~20%) of these CD68^+^ cells expressed CD163. RvD1 did not affect the number of intramuscular CD163^+^ macrophages but did reduce total macrophage numbers closer to numbers typically seen in uninjured muscle (Figure 6F).

### Resolvin D1 minimally affects the global MuSC transcriptome but may specifically modulate genes related to muscle-immune interactions.

**Figure 7:**
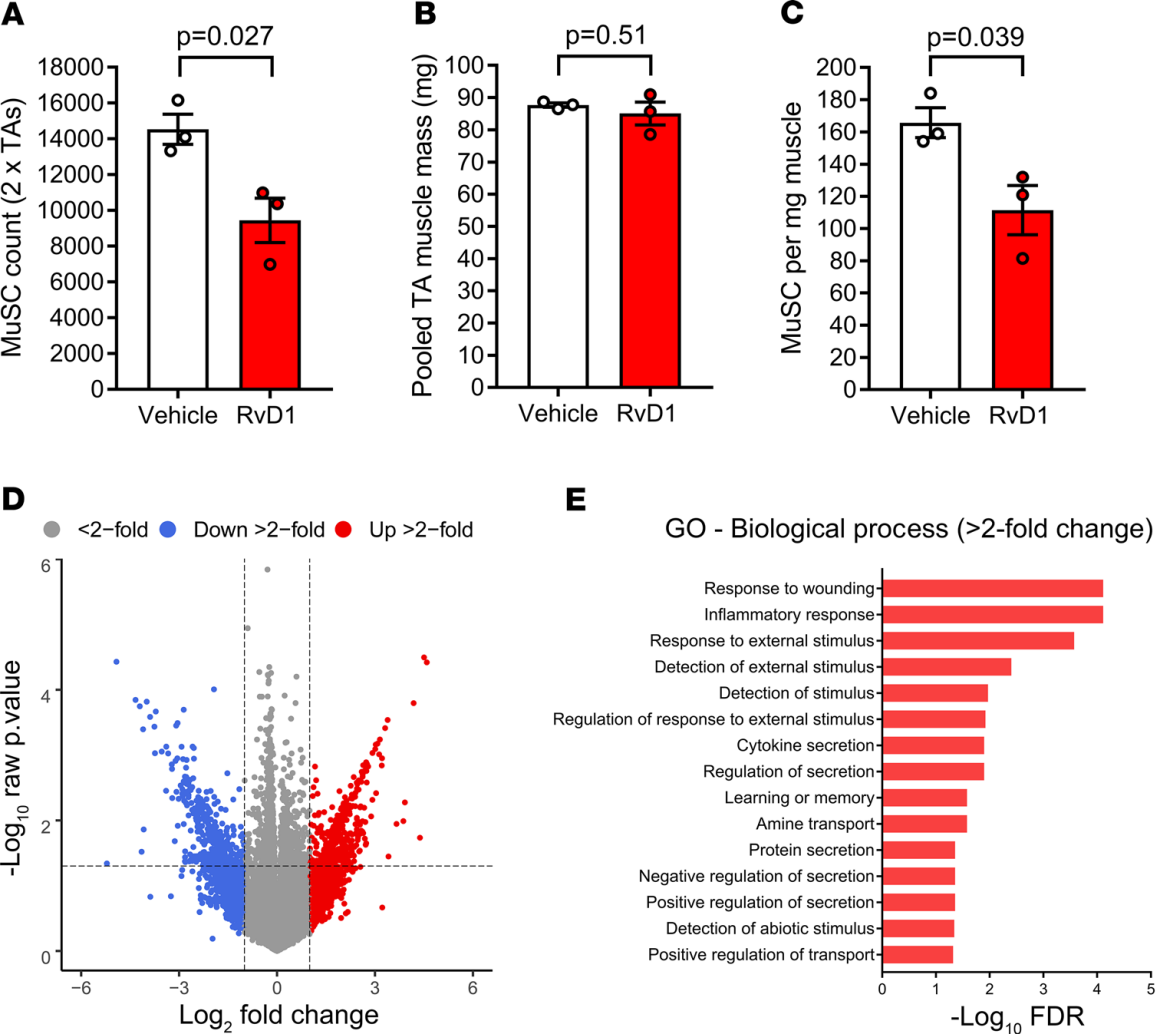
Resolvin D1 minimally affects the global transcriptomic response of muscle stem cells to injury but may modulate genes related to muscle-immune cell interactions. C57BL/6 mice received bilateral intramuscular injection of the TA muscle with 50 μL of 1.2% BaCl_2_ induce myofiber injury. Mice were treated with daily IP injection with RvD1 (100 ng) or vehicle control (0.1% ethanol) for 72 hours. Both TA muscles were collected at day 3 postinjury and pooled. Muscle stem cells (satellite cells, MuSCs) were isolated by FACS, and transcriptome-wide profiling of the isolated MuSC population was performed by RNA-Seq. (**A**) Total MuSC yield from day 3 postinjury TA muscles. (**B**) Pooled mass of TA muscles used for MuSC isolation. (**C**) Relative MuSC yield. (**D**) Volcano plot of overall RNA-Seq data with each dot representing a single gene, positive log_2_ fold change (FC) indicating induction, and negative log_2_ FC indicating suppression in response to RvD1 treatment. Genes induced more than 2 FC (+1 log_2_ FC) are colored red, and those suppressed more than 2 FC (-1 log_2_ FC) are colored blue. (**E**) Gene ontology enrichment and respective FDRs for genes up- or downregulated more than 2 FC (irrespective of *P* value) in response to RvD1. Bars show the mean ± SEM of 3 mice per group with dots representing data from each mouse. *P* values were determined 2-tailed unpaired *t* tests (**A**–**C**) or limma-voom differential expression analysis (**D**).

In order to gain insight into the mechanisms by which RvD1 enhanced muscle regeneration, we performed transcriptome-wide profiling of gene expression of MuSCs isolated from TA muscles day 3 after BaCl_2_ injection via FACS followed by RNA-sequencing (RNA-Seq). The absolute MuSC yield from injured TA muscles was lower in RvD1-treated mice (Figure 7A), while TA muscle mass was not affected (Figure 7B), indicating relative muscle MuSC number was reduced in RvD1-treated mice (Figure 7C). Overall, the global transcriptomic profile was very similar for isolated MuSCs from vehicle- and RvD1-treated mice at day 3 postinjury (Figure 7D) and differed markedly from uninjured MuSCs (Supplemental Figure 4A). No individual genes were detected as significantly differentially expressed between RvD1- and vehicle-treated MuSCs after controlling for the false discovery rate (FDR) (Benjamini-Hochberg adjusted *P* < 0.05). Nevertheless, assessment of gene lists based on magnitude of change (>2-fold) between RvD1- and vehicle-treated mice did reveal an enrichment of genes associated with response to wounding (76 genes), the inflammatory response (54 genes), response to external stimulus (98 genes), and cytokine secretion (17 genes), among others (Figure 7E). A heatmap of the 100 most differentially expressed annotated genes in isolated MuSCs between vehicle- and RvD1-treated mice is shown in Supplemental Figure 4B.

### Resolvin D1 neither stimulates nor perturbs in vitro myogenesis.

**Figure 8:**
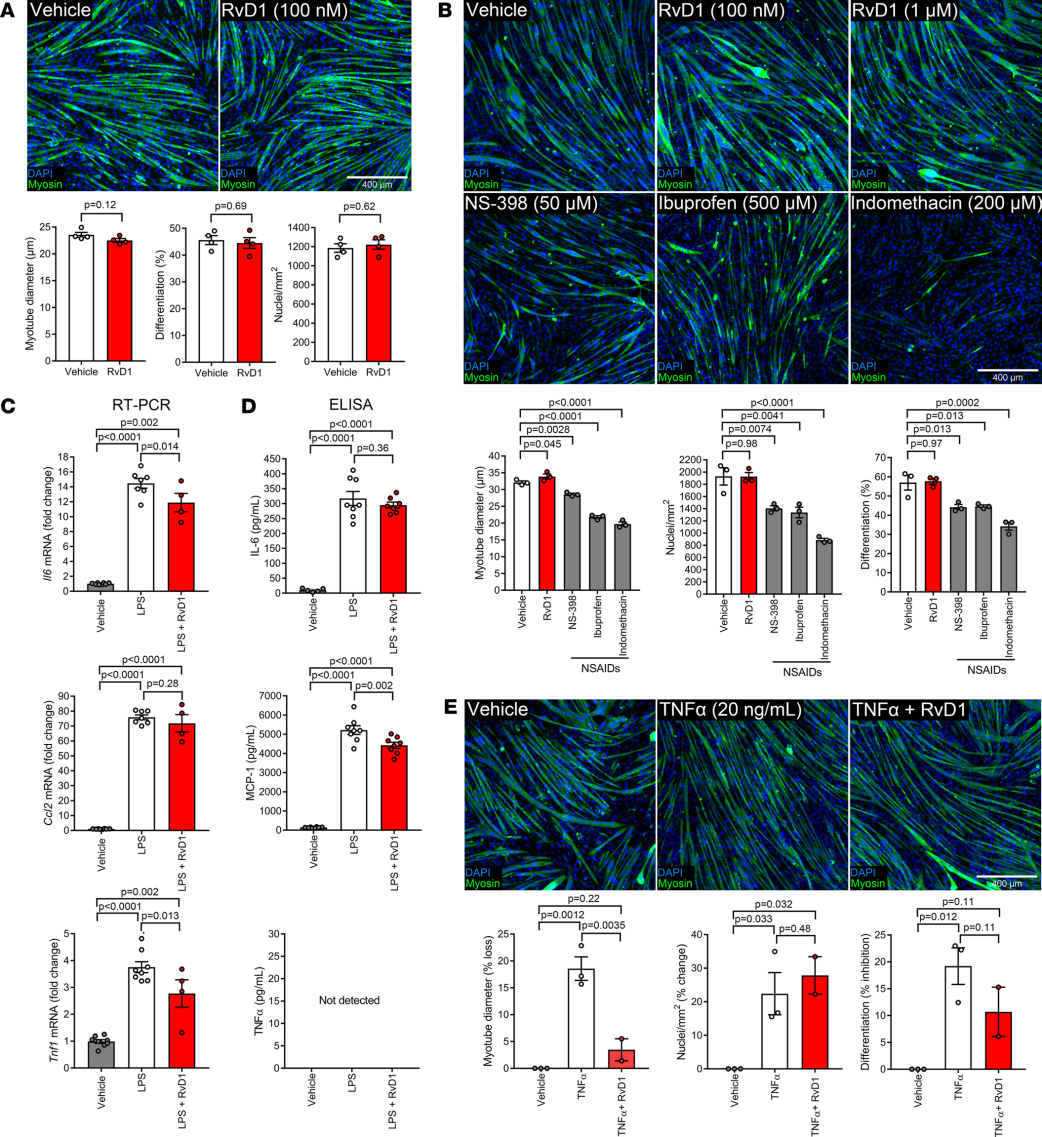
Resolvin D1 neither enhances nor perturbs basal in vitro myogenesis but suppresses myokine production and protects muscle cells against the deleterious effects of chronic inflammation. (**A**) Murine C2C12 myoblasts were treated with RvD1 (100 nM) at onset of myogenic differentiation. At 3 days postdifferentiation, myotubes were fixed in 4% paraformaldehyde (PFA) and stained for sarcomeric myosin. Cell nuclei were counterstained with DAPI. Quantitative analysis was performed on 6 nonconsecutive fields of view per well to determine overall cell density (DAPI^+^ nuclei/mm^2^), the extent of myogenic differentiation (% DAPI^+^ nuclei within myosin^+^ cells), and mean myotube (multinucleated cell) diameter. (**B**) Confluent C2C12 myoblasts were induced to differentiate in the presence of RvD1 (0.1–1 μM), or NSAIDs, including NS-398 (50 μM), ibuprofen (500 μM), and indomethacin (200 μM). (**C**) C2C12 myotubes at day 3 postdifferentiation were pretreated with RvD1 (100 nM) for 30 minutes before stimulation with lipopolysaccharide (LPS, 100 ng/mL) for 3 hours in the continued presence of RvD1. mRNA expression of cytokines, including IL-6, MCP-1, and TNF-α, was determined by RT-PCR. (**D**) C2C12 myotubes at day 3 postdifferentiation were pretreated with RvD1 (100 nM) for 30 minutes and then stimulated with LPS (100 ng/mL) for 24 hours in the continued presence of RvD1. Conditioned culture medium was collected from the cells and analyzed for concentrations of the cytokines IL-6, MCP-1, and TNF-α by ELISA. (**E**) Confluent C2C12 myoblasts were induced to differentiate in the presence of exogenous TNF-α (20 ng/mL), with or without RvD1 (100 nM) cotreatment. At day 3 postdifferentiation, myotubes were fixed, stained, and quantified as described in **A**. Scale bars: 400 μm. Bars show the mean ± SEM of 3–8 replicates per group with dot representing data for a single independent culture well. *P* values were determined by 2-tailed unpaired *t* tests (**A**) or 1-way ANOVA followed by pairwise Holm-Šidák post hoc tests (**B**–**E**).

We also tested whether RvD1 could directly influence muscle cell growth in vitro. A single dose of 100 nM RvD1, based on bioactivity in murine macrophages (Figure 4), at the time of myogenic differentiation did not influence overall cellular density (DAPI^+^ nuclei/mm^2^), myoblast differentiation (% DAPI^+^ nuclei within myosin^+^ cells), or fused myotube size (mean diameter) (Figure 8A). Because NSAIDs have direct suppressive effects on in vitro myogenesis (30), we questioned whether higher doses of RvD1 would also interfere with myogenesis. As previously reported, the nonspecific COX pathway inhibitors ibuprofen and indomethacin, as well as the COX-2 selective inhibitor NS-398, impaired myotube development in a dose-dependent manner (Supplemental Figure 5). Maximally effective doses of NS-398 (50 μM), ibuprofen (500 μM), and indomethacin (200 μM) reduced indices of myoblast density, percentage differentiation, and fused myotube diameter (Figure 8B). In contrast, muscle cells treated with RvD1 at doses of 0.1–1 μM showed no such deleterious effects, and a modest yet statistically significant increase in fused myotube size (+5%) was observed with 1 μM RvD1 (Figure 8B).

### Resolvin D1 directly limits myokine production and protects muscle cells under conditions of chronic inflammatory stress.

RvD1 can suppress cytokine production by a variety of cell types, most notably macrophages (31). Therefore, we questioned whether RvD1 could also directly modulate inflammatory cytokines in muscle cells (myokines). Exposure of C2C12 myotubes to lipopolysaccharide (LPS) markedly increased production of IL-6, MCP-1, and TNF-α at the gene and protein levels (Figure 8, C and D). Pretreatment with RvD1 blunted (3-hour) LPS-stimulated mRNA expression of IL-6 and TNF-α but did not influence MCP-1 (Figure 8C). RvD1 also reduced LPS-stimulated secretion of MCP-1 during more prolonged treatment (24 hours) (Figure 8D).

In order to determine whether RvD1 may directly influence myogenesis under conditions of chronic non-resolving inflammation, C2C12 myoblasts were also induced to differentiate in the presence of the proinflammatory cytokine TNF-α (20 ng/mL) (Figure 8E). Long-term TNF-α exposure reduced the number of myoblasts successfully undergoing myogenic differentiation and led to formation of thin, elongated myotubes. Cotreatment with RvD1 (100 nM) protected against the deleterious effect of TNF-α on developing myotube size but did not rescue TNF-α–induced effects on myoblast cellular density or myogenic differentiation (Figure 8E).

## Discussion

We investigated the role of SPMs in inflammatory and adaptive responses to muscle damage and tested the efficacy of daily systemic immunoresolvent treatment as a novel therapeutic strategy for treatment of muscular injuries. SPMs were locally produced in response to both chemical injury and functional overload. RvD1 suppressed muscle inflammatory cytokines, reduced M1-like macrophage infiltration, expedited tissue PMN clearance, and shifted intramuscular macrophages to a more M2-like activation state. These immunomodulatory effects were associated with enhanced regenerating myofiber growth and improved recovery of muscle strength. While RvD1 had little direct impact on myogenesis in vitro, it suppressed myokine production and enhanced macrophage phagocytosis indicative of both muscle direct and indirect actions. These findings show that SPMs play important supportive roles in myofiber regeneration and highlight their therapeutic potential in the context of muscle injuries as a novel alternative to classic antiinflammatory interventions (e.g., NSAIDs), which are known to inhibit endogenous cellular regenerative mechanisms.

Intramuscular injection of BaCl_2_ results in widespread myofiber necrosis and a robust inflammatory response. In this model, restoration of muscle function is dependent on de novo muscle fiber formation via the proliferation, differentiation, and fusion of MuSCs (myofiber regeneration). In contrast, functional muscle overload induced by synergist ablation results in mild myofiber damage, and ensuing adaptive tissue remodeling occurs mainly from compensatory growth of existing muscle cells (myofiber hypertrophy). We show here that both interventions resulted in rapid infiltration of muscle by PMNs, their subsequent disappearance, and a persistent intramuscular macrophage presence. In both models, an initial increase in expression of inducible COX-2 led to increased intramuscular concentrations of proinflammatory prostaglandins (e.g., PGE_2_), which have been implicated in stimulating skeletal muscle regeneration (32). PGE_2_ also plays a key role in initiating the resolution phase of inflammation, inducing transcription of 15-LOX (33). Consistent with this concept, expression of 5-, 12-, and 15-LOX, as well as intramuscular concentrations of LOX-derived SPM pathway markers, increased during muscle regeneration. COX-2 exhibited a bimodal response, consistent with a dual role of prostaglandins in both induction and resolution of acute inflammation (34). We also showed for the first time to our knowledge heightened local 5- and 15-LOX expression and increased tissue abundance of many SPM pathway markers following functional overload of the rat plantaris muscle.

Bioactive SPMs themselves were generally not present at detectable concentrations in uninjured muscle, but intramuscular protectin D1 and maresin 1 were detected following both BaCl_2_ injury and functional overload, while resolvin D6 and lipoxin A_4_ were also detected in the latter model. SPMs or their pathway markers increase in mouse muscle following limb ischemia (18), intramuscular cardiotoxin injection (19), or eccentric exercise (19), and in human muscle biopsies following damaging muscular contractions (17). Acute exercise stress also transiently increases SPMs in human blood (16), and repeated exercise training primes murine macrophages to release greater amounts of RvD1 in response to an inflammatory challenge (35). PMNs, inflammatory monocytes, and reparative macrophages isolated from cardiotoxin-injured mouse muscle also display distinct time-dependent modulation of lipid mediators (19). Overall, these findings show that dynamic regulation of the SPMs is conserved across multiple species and models of muscle damage and/or stress.

We could not detect endogenous RvD1 in muscle but did observe increased expression of the enzymatic machinery for RvD1 biosynthesis (15- and 5-LOX) (24) and 17-HDoHE, the primary 15-LOX intermediate product of DHA produced during D-series resolvin biosynthesis (25) following injury. We have previously shown increased circulating RvD1 following strenuous exercise (16). Notably, primary LOX metabolites (e.g., 17-HDoHE) were far more abundant in muscle homogenates in the current study than bioactive SPMs themselves. This suggests that the lipid metabolome of muscle may predominantly reflect intracellular metabolites whereas bioactive SPMs may be relatively enriched in the extracellular environment, where they bind to their cell surface receptors (26). Another possibility is that bioactive SPMs in tissues are rapidly converted to downstream enzymatic inactivation products. Consistently, we observed 8-oxo-RvD1 (a bioactive 15-hydroxy prostaglandin dehydrogenase metabolite of RvD1, ref. 25) in rat muscle 3 days after synergist ablation at levels ~2-fold higher than baseline. Nevertheless, Sansbury et al. 2020 reported that RvD1 is indeed present within mouse skeletal muscle tissue, at very low concentrations (36).

Blockade of PMN influx following muscle damage protects against leukocyte-induced secondary injury (4). Therefore, limiting PMN recruitment and hastening PMNs’ removal have been proposed to be organ protective in skeletal muscle injury. A class-defining action of SPMs is the selective inhibition of further PMN recruitment to the site of inflammation (23). Indeed, systemic injection of resolvins displays similar potency in reducing PMN infiltration in the murine peritonitis model as the NSAID indomethacin (23). On this basis, we hypothesized that treatment with RvD1 at the time of BaCl_2_ injury would limit the initial appearance of PMNs, but the peak intramuscular PMN response was unchanged by the treatment. RvD1 reduced intramuscular PMNs at day 3 of recovery, however. Consistent with our findings, prior studies showed no reduction in response to RvD1 of peak PMN infiltration of cardiac muscle following myocardial infarction but an accelerated PMN egress (37, 38). Overall, these data are consistent with the notion that in addition to their antiinflammatory actions, SPMs actively promote PMN clearance, thus accelerating resolution to a noninflamed state (39). One such mechanism is the ability of SPMs to stimulate macrophage-mediated phagocytic uptake and removal of apoptotic PMNs (40). Consistent with prior studies (e.g., refs. 26, 28), RvD1 proved a potent stimulator of macrophage phagocytic activity in our hands. Notably, traditional antiinflammatory drugs such as NSAIDs, which lack the proresolving bioactivity that we and others have observed for SPMs, can delay timely resolution of inflammation by limiting monocyte recruitment and interfering with macrophage-mediated clearance of tissue PMNs (34).

One key molecular mechanism by which resolvins function is through blunting production of proinflammatory cytokines (23). We found that systemic treatment with RvD1 at the time of muscle injury suppressed local cytokine expression. RvD1 also directly reduced LPS-stimulated mRNA expression of IL-6 and TNF-α, as well as secretion of MCP-1 protein by muscle cells cultured in vitro. Resolvin E1 (RvE1) also directly inhibits cytokine production by muscle cells (41). While the overall impact of systemic RvD1 treatment on the global MuSC transcriptome following injury was minimal in the current study, clusters of genes with strong relevance to muscle-immune interactions such as those involved in the response to wounding, the inflammatory response, and cytokine secretion were among those RvD1 influenced most. Collectively, these data suggest that resolvins have some direct modulatory effects on the production of inflammatory mediators by cells resident to the musculature, such as postmitotic myofibers and resident MuSCs.

MCP-1 is indispensable for recruitment of blood monocytes to injured muscle (42). Given the suppressive effect of RvD1 on muscle MCP-1 expression, the initial reduction observed in the current study in intramuscular macrophages following RvD1 treatment is not surprising. Recovery of muscle macrophage numbers to control levels during the resolution phase suggests that RvD1 treatment may either stimulate later monocyte recruitment or promote expansion of tissue macrophage populations. Indeed, mice receiving RvD1 also displayed heightened muscle expression of the alternative macrophage activation marker Arg-1 and greater intramuscular macrophages expressing the M2-like marker CD163. Overall, these data are consistent with prior studies showing that RvD1 can induce an M2-like polarization state of macrophages in vitro (43). In turn, M2-polarized macrophages produce more SPMs than M1-activated macrophages (44), which may explain why muscle expression of 12-LOX, a key SPM biosynthetic enzyme expressed by macrophages, was increased in muscle in response to RvD1 treatment here. While most M1 signature cytokines did not differ between vehicle- and RvD1-treated mice during the regenerative phase, IL-1β, a classical proinflammatory cytokine, was ~3-fold higher in muscle of RvD1-treated mice at day 5 postinjury. This may potentially be explained by recent studies showing that resolution phase macrophages are neither classically nor alternatively activated but possess certain aspects of both phenotypes (45).

NSAIDs can have deleterious effects on muscle repair (12). Because SPMs also possess some antiinflammatory actions, it was important to determine whether repeated daily immunoresolvent treatment affects the efficiency of muscle regeneration. Overall, we found no evidence of a deleterious effect of RvD1 on any indices of myofiber regeneration in vivo. Moreover, the well-established direct suppressive effects of NSAIDs on in vitro myogenesis were clearly not shared by RvD1, even at high doses (30). Rather, RvD1 enhanced muscle repair in mice in vivo as evidenced by increased regenerating muscle fiber size and improved recovery of muscle strength. Additionally, RvD1 (1 μM) directly promoted modest, yet statistically significant, hypertrophy of developing myotubes in vitro, consistent with prior studies showing that nonmyeloid cell types (e.g., fibroblasts) may be more responsive to relatively higher doses of RvD1 (46). Nevertheless, lower doses of RvD1 (100 nM) could also protect muscle cells from the deleterious effects of chronic exposure to proinflammatory stimuli, as previously shown with RvE1 (41). The contrasting effects of NSAIDs and RvD1 on muscle regenerative capacity may be explained by their varying mechanisms of action. Whereas NSAIDs decrease intramuscular macrophage infiltration (29), impair M2 polarization (47), and suppress macrophage phagocytosis (40), RvD1 had overall stimulatory effects on macrophage responses. Macrophages in general, and M2-like macrophages in particular, are thought to support muscle regeneration (1). Therefore, the beneficial effect of RvD1 on muscle regeneration may be attributable to direct positive interactions between intramuscular macrophages and resident myofibers and/or MuSCs. Phagocytosis of muscle cell debris is a key stimulatory cue triggering macrophage polarization (2), and blocking phagocytosis has deleterious effects on both intramuscular macrophage phenotype transitions and myofiber regeneration (3). Therefore, the stimulatory effects of RvD1 on phagocytosis, intramuscular macrophage activity, and myofiber regeneration are likely complex and intrinsically linked.

Intramuscular injection of RvD2, a related but structurally distinct SPM, which acts via a different cell surface receptor (GPR18), was recently reported to improve recovery of muscle mass and strength following cardiotoxin-induced muscle injury in mice (19). That study focused primarily on immunological outcomes, and the underlying cellular and molecular basis for the apparent effects on muscle regeneration were not investigated. Moreover, before muscle injury, mice were depleted of resident MuSCs by irradiation, which is known to deregulate the normal inflammatory and regenerative responses to muscle injury, as further evidenced by the marked weakness at day 14 postinjury when compared with the healthy young mice in the present study (900 vs. 200 mN P_o_) (19). Under the physiologically relevant circumstances in which MuSCs play a well-established and indispensable role in de novo myofiber formation (20), we observed that the beneficial effects of RvD1 on recovery of muscle size and strength were mechanistically attributable to cellular hypertrophy of regenerating myofibers, independent of any change in the overall extent of new myofiber formation. We further show an impact of immunoresolvent treatment on intramuscular PMN clearance from injured muscle, which provides one more mechanism that may explain the therapeutic benefit of immunoresolvents in the context of muscle regeneration (4). In support of our findings, annexin A1, a distinct agonist of the RvD1 receptor (FPR2/ALX), was also recently shown to be required for effective myofiber regeneration in healthy young mice (21, 22).

The demonstrated benefit of systemic immunoresolvent treatment on muscle inflammation and regeneration has important implications for future therapeutic translation to human studies, especially given that RvD1 is orally bioavailable in mice (48). Moreover, the ability of daily dosing with RvD1 throughout the entire time course of recovery from injury to ultimately improve muscle regeneration rather than compromise it, as is the case with daily systemic NSAID treatment, is in itself novel (12). This clear advantage of RvD1 treatment compared with NSAIDs is of great clinical importance given that frequent repeated dosing would likely be required to effectively exploit the analgesic potential of SPMs for pain management, which is a key therapeutic goal in the clinical treatment of soft-tissue injuries (49). We chose to administer RvD1 preemptively at the time of injury and then daily throughout recovery from muscle damage in the current study so as to be directly comparable with the prophylactic dosing regimens of prior studies showing a deleterious effect of NSAIDs on muscle regeneration in mice (29). Therefore, it will be important for future studies to also test whether delayed RvD1 treatment has similar beneficial effects on muscle regeneration.

A further potentially novel finding of the current study is the demonstration for the first time that SPM biosynthetic circuits are locally induced in response to overload-induced muscle hypertrophy. In addition to their key supportive role in muscle regeneration, macrophages regulate overload-induced myofiber hypertrophy (50) and myofiber regrowth during recovery from disuse (51). Thus, our observation of SPM biosynthesis in muscle with increased load suggests that immunoresolvents may also have potential therapeutic applicability to modulate adaptive responses to functional unloading and loading of muscle. Therefore, further studies should address the effects of immunoresolvents on adaptive remodeling of postmitotic myofibers, such as overload-induced muscle hypertrophy as well as atrophy/regrowth induced by muscle disuse and subsequent reloading. Immunoresolvents may also be an effective novel therapeutic in physiologically and clinically relevant settings characterized by sustained, nonresolving, acute inflammatory responses and limited regenerative efficiency, such as volumetric muscle loss (52). Finally, states of chronic unresolved inflammation that negatively affect muscle mass and myofiber regenerative capacity, including aging (53), muscular dystrophies (11), and metabolic disease (e.g., obesity/diabetes) (54), may also benefit from this therapeutic strategy and should be investigated.

One potential limitation of the current study is the use of only female mice. A recent study revealed sex-related differences in SPM biosynthesis following myocardial infarction, with male mice producing relatively more endogenous SPMs than females (55). Thus, immunoresolvents may be a relatively more effective therapeutic in females than males. Nevertheless, Giannakis et al. 2019 found a benefit of RvD2 treatment on skeletal muscle regeneration in male mice also (19). Future studies investigating the impact of sex on endogenous lipid mediator responses to skeletal muscle injury and the efficacy of immunoresolving therapies will be valuable for future development of personalized musculoskeletal medicine.

In conclusion, SPMs are locally produced in response to myofiber damage induced by both degenerative injury and physiological muscle loading. Daily systemic treatment with RvD1 modulates muscle inflammation, expedites its active resolution, and enhances cellular transitions of macrophages, MuSCs, and myofibers to enable effective muscle tissue repair. These data implicate endogenous SPMs in controlling adaptive muscle remodeling in healthy young mice and highlight the potential efficacy of systemic immunoresolving therapies as a novel treatment of muscle injuries that can enhance regeneration by stimulating endogenous resolution mechanisms.

## Methods

### Animals.

C57BL/6 mice and Sprague-Dawley rats were obtained from Charles River Laboratories and housed under specific pathogen–free conditions with ad libitum access to food and water.

### Muscle injury.

Female C57BL/6 mice (4–6 months) were anesthetized with 2% isoflurane and received bilateral intramuscular injection of the TA muscle with 50 μL per limb of 1.2% BaCl_2_ in sterile saline. Age- and sex-matched mice were randomized to receive sham injury via intramuscular injection of sterile saline alone instead of BaCl_2_. Mice were returned to their home cage to recover and monitored until ambulatory. Between 5 and 10 mice were randomly allocated to each experimental group with each individual mouse considered a biological replicate. The exact number of biological replicates for each specific experiment is indicated within the relevant figure legend.

### Functional muscle overload.

Myotenectomy-induced synergist ablation was used to assess the inflammatory response to functional overload of the plantaris muscle as originally described by Goldberg et al. 1967 (56). Male 6-month-old rats were anesthetized with 2% isoflurane, and preemptive analgesia was provided by subcutaneous injection of buprenorphine (0.03 mg/kg) and carprofen (5 mg/kg). The skin overlying the posterior hind limb was shaved and scrubbed with chlorhexidine and ethyl alcohol. A midline incision was made to visualize the gastrocnemius/soleus (Achilles) tendon and a full-thickness tenectomy performed while leaving the plantaris tendon intact. The incision was closed using 4–0 Vicryl sutures. The procedure was then repeated on the contralateral limb to induce bilateral functional overload of both plantaris muscles. Rats were returned to their cage to recover and monitored until ambulatory with free access to food and water. Postoperative analgesia was provided via subcutaneous injection of buprenorphine (0.03 mg/kg) at 12 hours postsurgery, and animals were monitored daily for any signs of pain or distress for 7 days. Age- and sex-matched rats served as nonsurgical controls. Between 4 and 6 rats were randomly allocated to each experimental group with each limb considered a biological replicate. The number of biological replicates for each experiment is indicated within figure legends.

### Immunoresolvent treatment.

RvD1 was purchased from Cayman Chemicals (10012554). Single-use aliquots of RvD1 were prepared in amber glass vials (Thermo Fisher Scientific, C4010-88AW), which were purged with nitrogen gas and stored at –80°C. On the day of use, the ethanol was evaporated to dryness under a gentle stream of nitrogen gas, and RvD1 was resuspended in sterile saline containing 0.1% ethanol. RvD1 stocks were handled in a dark room, and aqueous solutions of RvD1 were protected from light and used within 30 minutes of preparation. Female C57BL/6 mice (4–6 months) were randomized to receive daily 100 μL IP injections of either 100 ng of RvD1 or vehicle control (0.1% ethanol), with the first dose administered approximately 5 minutes before muscle injury. Mice were allowed to recover for up to 2 weeks postinjury with daily IP injection of 100 ng of RvD1 or vehicle.

### Muscle tissue collection.

Animals were euthanized via induction of bilateral pneumothorax while under isoflurane anesthesia. Muscles were rapidly dissected, weighed, and snap-frozen in liquid nitrogen. Muscles for histological analysis were cut transversely at the midbelly with a scalpel blade, oriented longitudinally on a plastic support, covered with a thin layer of optimal cutting temperature compound, and rapidly frozen in isopentane cooled on liquid nitrogen. Samples were stored at –80°C until analysis.

### Histological analysis of muscle inflammation and regeneration.

Tissue cross sections (10 μm) were cut from the muscle midbelly in a cryostat at –20°C and adhered to SuperFrost Plus slides. Sections were air-dried and then stained with H&E. Slides for immune cell staining were fixed in acetone for 10 minutes at –20°C and then air-dried. MuSC slides were fixed in 4% PFA for 15 minutes at room temperature and quenched with hydrogen peroxide, and heat-mediated antigen retrieval was performed. Unfixed tissue sections were used for muscle fiber type staining. Prepared slides were blocked in 10% normal goat serum (Invitrogen, Thermo Fisher Scientific, 10000C) or Mouse on Mouse blocking reagent (VECTOR Laboratories, MKB-2213) before overnight incubation at 4°C with primary antibodies. The following day, slides were incubated with appropriate secondary antibodies and mounted using Fluorescence Mounting Medium (Agilent Dako, S302380). Fluorescent images were captured using a Nikon A1 confocal microscope.

### Immunohistochemistry antibodies.

Primary antibodies used on mouse muscle included eMHC (Developmental Studies Hybridoma Bank [DSHB], F1.652s, 1:20), Pax7 (DSHB, Pax7c, 1:100), MHC I (DSHB, BA-D5c, 1:100), MHC IIa (DSHB, SC-71c, 1:100), MHC IIb (DSHB, BF-F3c, 1:100), laminin (Abcam, ab7463, 1:200), Ly6G (Gr-1) (BD Biosciences, BD550291, 1:50), CD68 (Bio-Rad, MCA1957, 1:50), and CD163 (Santa Cruz Biotechnology, sc-33560, 1:50). Primary antibodies used on rat muscle included MHC type I (DSHB, BA-D5c, 1:100), MHC IIa (DSHB, SC-71c, 1:100), MHC IIb (DSHB, BF-F3c, 1:100), HIS48 (Abcam, Ab33760, 1:20), CD68 (Abcam, ab31630, 1:50), and CD163 (Santa Cruz Biotechnology, sc-33560, 1:50). Antibody binding was visualized with standard Alexa Fluor secondary antibodies (Invitrogen, Thermo Fisher Scientific, 1:500 in PBS), except for Pax7, which was detected using a Tyramide SuperBoost Kit (Invitrogen, Thermo Fisher Scientific B40913). Fluorescent dyes DAPI (Invitrogen, Thermo Fisher Scientific, D21490, 2 μg/mL), wheat germ agglutinin (WGA) Alexa Fluor 647 conjugate (Invitrogen, Thermo Fisher Scientific, W32466, 5 μg/mL), WGA CF405S conjugate (Biotium, 29027, 100 μg/mL), and phalloidin (Invitrogen, Thermo Fisher Scientific ActinRed 555 ReadyProbes, R37112) were used to counterstain cell nuclei, extracellular matrix, and muscle fibers, respectively.

### Image analysis.

Muscle tissue morphology was analyzed on stitched panoramic images of the entire muscle cross section by high-throughput fully automated image analysis with the MuscleJ plugin for FIJI (57), with few exceptions. At day 5 after BaCl_2_ injury, regenerating (eMHC^+^) fiber number and size were quantified on the stitched panoramic images of the entire TA muscle cross section by in-house semiautomated image analysis using ImageJ/FIJI (Supplemental Figure 3). Immune cells were manually counted throughout the entire mouse TA cross section and normalized to total tissue area as determined by MuscleJ or from 5 nonoverlapping ×20 fields of view captured from within the inflammatory lesion of the rat plantaris (Supplemental Figure 2). In all cases, the experimenter was blinded to the experimental group.

### Muscle force testing.

Mice were anesthetized with 2% isoflurane and placed on a heated platform. The distal half of the TA muscle was isolated by dissecting the overlying skin and fascia. The knee joint was immobilized and a 4–0 silk suture tied around the distal TA tendon, which was cut and tied to the lever arm of a servomotor (6650LR, Cambridge Technology). A saline drip warmed to 37°C was continuously applied to the exposed muscle. The peroneal nerve was stimulated with 0.2 ms pulses using platinum electrodes with the stimulation voltage and muscle length adjusted to obtain optimal muscle length (L_o_) and maximum isometric twitch force (P_t_). The muscle was then stimulated at increasing frequencies while held at L_o_ until maximum isometric tetanic force (P_o_) was achieved. A 1-minute rest was allowed between each tetanic contraction. Muscle length was measured with calipers and optimum fiber length (L_f_) determined by multiplying L_o_ by the TA muscle L_f_/L_o_ ratio of 0.6 (58). The CSA of the muscle was calculated by dividing muscle mass by the product of L_f_ and 1.06 mg/mm^3^ (59). sP_o_ was calculated by dividing P_o_ by muscle CSA.

### LC-MS/MS–based metabolipidomic profiling of muscle tissue.

Muscle samples were mechanically homogenized in 1 mL PBS using a bead mill. The tissue homogenates were centrifuged at 3000*g* for 5 minutes and the supernatant was collected. Supernatants (0.85 mL) were spiked with 5 ng each of 15(S)-HETE-d8, 14(15)-EpETrE-d8, Resolvin D2-d5, Leukotriene B4-d4, and Prostaglandin E1-d4 as internal standards (in 150 μL methanol) for recovery and quantitation and mixed thoroughly. The samples were then extracted for polyunsaturated fatty acid metabolites using C18 extraction columns as previously described (16, 17). Briefly, the internal standard spiked samples were applied to conditioned C18 cartridges, washed with 15% methanol in water followed by hexane, and then dried under vacuum. The cartridges were eluted with 2 volumes of 0.5 mL methanol with 0.1% formic acid. The eluate was dried under a gentle stream of nitrogen. The residue was redissolved in 50 μL methanol–25 mM aqueous ammonium acetate (1:1) and subjected to LC-MS/MS analysis.

HPLC was performed on a Prominence XR system (Shimadzu) using Luna C18 (3 μm, 2.1 × 150 mm) column. The mobile phase consisted of a gradient between A, methanol-water-acetonitrile (10:85:5 v/v), and B, methanol-water-acetonitrile (90:5:5 v/v), both containing 0.1% ammonium acetate. The gradient program with respect to the composition of B was as follows: 0–1 minute, 50%; 1–8 minutes, 50%–80%; 8–15 minutes, 80%–95%; and 15–17 minutes, 95%. The flow rate was 0.2 mL/min. The HPLC eluate was directly introduced to the electrospray ionization source of a QTRAP 5500 mass analyzer (ABSCIEX) in the negative ion mode with following conditions: curtain gas: 35 psi, GS1: 35 psi, GS2: 65 psi, temperature: 600°C, ion spray voltage: –1500 V, collision gas: low, declustering potential: –60 V, and entrance potential: –7 V. The eluate was monitored by Multiple Reaction Monitoring (MRM) method to detect unique molecular ion–daughter ion combinations for each of the lipid mediators using a scheduled MRM around the expected retention time for each compound. Optimized collisional energies (18–35 eV) and collision cell exit potentials (7–10 V) were used for each MRM transition. Spectra of each peak detected in the scheduled MRM were recorded using enhanced product ion scan to confirm the structural identity. The data were collected using Analyst 1.7 software, and the MRM transition chromatograms were quantitated by MultiQuant software (both from ABSCIEX). The internal standard signals in each chromatogram were used for normalization, recovery, as well as relative quantitation of each analyte.

LC-MS/MS data were analyzed using MetaboAnalyst 4.0 (60). Analytes with more than 50% missing values were removed from the data set, and remaining missing values were replaced with half of the minimum positive value in the original data set. Heatmaps were generated using the Pearson distance measure and the Ward clustering algorithm following autoscaling of features without data transformation. Targeted statistical analysis was performed on a predetermined subset of metabolites of interest.

### Whole-tissue RNA extraction and RT-qPCR.

Muscle was homogenized in TRIzol reagent using a bead mill. RNA was isolated by phenol/chloroform extraction and RNA yield determined using a NanoDrop Spectrophotometer (NanoDrop 2000c, Thermo Fisher Scientific). Genomic DNA was removed by incubation with DNase I (Ambion, Thermo Fisher Scientific, AM2222) followed by its heat inactivation. Total RNA (1 μg) was reverse-transcribed to cDNA using SuperScript VILO Master Mix (Invitrogen, Thermo Fisher Scientific 11-755-050) and RT-qPCR performed on a CFX96 Real-Time PCR Detection System (Bio-Rad, 1855195) in duplicate 20 μL reactions of iTaq Universal SYBR Green Supermix (Bio-Rad, 1725124) with 1 μM forward and reverse primer. Relative mRNA expression was determined using the 2^-ΔΔCT^ method with *Actb* and *Gapdh* serving as endogenous controls from mouse and rat samples, respectively. Primer sequences are listed in Table 1.

### Flow cytometry analysis of muscle inflammation.

Both TA muscles from 1 mouse were pooled, finely minced, and digested for 60 minutes at 37°C for in Hanks’ balanced salt solution lacking calcium and magnesium (HBSS^–/–^, Thermo Fisher Scientific), supplemented with 250 U/mL collagenase II (Thermo Fisher Scientific), 4.0 U/mL dispase (MilliporeSigma), and 2.5 mmol/L CaCl_2_ (MilliporeSigma). The resulting digest solution was filtered through a 40 μm cell strainer and centrifuged at 350*g* for 5 minutes. Cells were Fc blocked with a CD16/CD32 antibody (Thermo Fisher Scientific, 14-0161-82) for 10 minutes at 4°C and then incubated for 30 minutes on ice with primary antibodies, including including CD45-PE (30-F11) (Thermo Fisher Scientific, 12-0451-82), Ly-6G-FITC (1A8) (BD Pharmingen, 551460), CD64-APC (X54-5/7.1) (BioLegend, 139306, CD11c-APCe780 (N418) (BioLegend, 47-0114-80), and Live/Dead fixable violet (Thermo Fisher Scientific, L34963). Flow cytometry was performed using LSRFortessa cell analyzer (BD Biosciences) and data were analyzed with FlowJo software.

### FACS enrichment of muscle satellite cells for RNA-Seq.

Both TA muscles from 1 mouse were pooled, finely minced, and digested for 60 minutes at 37°C in DMEM with 0.2% (~5500 U/mL) collagenase type II and 2.5 U/mL dispase II. The resulting digest solution was filtered through a 70 μm cell strainer and then centrifuged at 350*g* for 5 minutes. Cells were resuspended and incubated for 30 minutes on ice with primary antibodies, including Sca-1-APC (BioLegend clone D7; 108112), CD45-AF488 (BioLegend clone 30-F11; 103121), CD11b-APC (BioLegend clone M1/70; 101212), Ter119-APC (BioLegend clone TER-119; 116212), CD29/β1-integrin-PE (BioLegend clone HMb-1; 102208), and CD184/CXCR-4-BIOTIN (BD Biosciences lot 6336587; 551968). Cells were then washed, centrifuged, and resuspended in PECy7-STREPTAVIDIN secondary antibody (eBioscience, Thermo Fisher Scientific, lot 4290713; 25-4317-82). The stained cells were filtered through a 35 μm cell strainer, propidium iodide viability dye was added (1 μg) (Thermo Fisher Scientific), and FACS was performed using a BD FACSAria III Cell Sorter (BD Biosciences). MuSCs, classified here as APC/FITC (Sca-1, CD11b, Ter119, CD45) double-negative and PE/PECy7 (β1-integrin/CXCR-4) double-positive cells, were sorted into TRIzol reagent (Thermo Fisher Scientific) and stored at –80°C.

### RNA-Seq.

RNA was extracted from FACS-sorted MuSCs using the miRNeasy Micro Kit (QIAGEN, 217084). RNA concentration and integrity were measured with a NanoDrop spectrophotometer (NanoDrop 2000c) and Bioanalyzer High Sensitivity RNA Chip (Agilent 2100). High-quality RNA (10 ng, RNA integrity number > 9) was used to produce cDNA libraries using SmartSeq v4 (Clontech, 634888). cDNA was prepared into sequencing libraries using 150 pg of full-length cDNA amplicons with the Illumina Nextera XT DNA Library Preparation Kit with dual index barcodes. Barcoded libraries were pooled and sequenced on an Illumina NextSeq 550 using 76-bp single-ended reads (Gene Expression Omnibus GSE146785).

### RNA-Seq data processing and analysis.

Single-stranded RNA-Seq data were aligned to the mm10 reference genome with the STAR algorithm (STAR_2.5.0a) and RSEM quantification applied to the aligned reads. Differentially expressed genes were identified in R using limma-voom analysis. Expected counts were first voom transformed to log_2_ cpm, and then surrogate variable analysis was performed with the SVA package. Surrogate variables were quantified and removed from the data matrix before linear modeling and differential expression analysis using the limma pipeline. Thresholds of adjusted *P* < 0.05 and log_2_ FC ≥ 1 were used to call differential genes. Gene enrichment analysis was assessed using NetworkAnalyst 3.0 (61).

### Muscle cell culture.

C2C12 myoblasts (ATCC, CRL-1772) were grown at 37°C, 5% CO_2_ in DMEM (Gibco, Thermo Fisher Scientific, 11995-073), supplemented with 10% FBS (Corning, MT35015CV) and antibiotics (penicillin 100 U/mL, streptomycin 100 μg/mL) (Gibco, Thermo Fisher Scientific 15140122). Myoblasts were plated at a cell density of 2.5 × 10^4^/cm^2^ in 12-well plates in growth media and allowed to proliferate and crowd for 72 hours. Confluent myoblasts were then switched to differentiation media consisting of DMEM (Gibco, Thermo Fisher Scientific, 11995-073) supplemented with 2% horse serum (Gibco, Thermo Fisher Scientific) and antibiotics. In some experiments C2C12 cells were treated with 100 ng/mL LPS (MilliporeSigma, L2630) or 20 ng/mL of recombinant mouse TNF-α (Gibco, Thermo Fisher Scientific, PMC3014) in the presence or absence of exogenous RvD1 treatment. Experimental treatments were prepared in differentiation media immediately before use and provided to cells either at the onset of myoblast differentiation or to fused myotubes at day 3 postdifferentiation. To assess cellular morphology, myotubes were fixed in 4% PFA, permeabilized with 0.2% Triton X-100, and blocked in 1% BSA before overnight incubation at 4°C with a sarcomeric myosin antibody (DSHB, MF 20s, 1:20). The following day, cells were incubated with a Goat Anti-Mouse IgG (H + L) Alexa Fluor 488–conjugated secondary antibody (Invitrogen, Thermo Fisher Scientific A28175) and DAPI to counterstain cell nuclei (Invitrogen, Thermo Fisher Scientific D21490, 2 μg/mL). Stained cells were visualized with a Nikon A1 inverted confocal microscope and fluorescent images captured at original magnification ×10 throughout a 5 × 5 field grid encompassing an area of 30 mm^2^ surrounding the center of the culture cell. Six nonconsecutive images from the same coordinates within this grid were randomly selected for manual analysis using ImageJ/FIJI.

### ELISA analysis.

Cytokine concentrations in C2C12 conditioned media (cell culture supernatants) were analyzed for IL-6 (R&D Systems, Bio-Techne, RDY406-05), MCP-1 (CCL2) (R&D Systems, Bio-Techne, DY479-05), and TNF-α (R&D Systems, Bio-Techne, DY410-05) following the manufacturer’s recommendations.

### Murine BMMs.

Bone marrow was collected from tibias and femurs of 4- to 6-month-old female C57BL/6 mice and cultured for 7 days at 37°C and 5% CO_2_ in DMEM supplemented with 10% FBS, antibiotics, and 20 ng/mL recombinant murine GM-CSF (BioLegend, 576304). Cells were then washed with PBS to remove nonadherent cells, and adherent BMMs were detached by incubation in TrypLE Select at 37°C (Gibco, Thermo Fisher Scientific, 12563011) followed by gentle cell scraping. BMMs were plated into 96-well plates at 1 × 10^5^ cells/well in growth media lacking GM-CSF and allowed to adhere overnight before use.

### Human PBMC-derived macrophages.

Venous blood was collected from human volunteers by venipuncture into EDTA vacutainers. PBMCs were isolated by density gradient centrifugation using Lympholyte-poly Cell Separation Media (Cedarlane, CL5071). Monocytes were then purified from isolated PBMCs by negative selection using a Human Monocyte Isolation Kit (STEMCELL Technologies, 19359) and cultured for 7 days in RPMI 1640 medium (Gibco, Thermo Fisher Scientific, 11875119) with 10% FBS, antibiotics, and human recombinant GM-CSF (20 ng/mL) (R&D Systems, Bio-Techne, 215-GM-010). Cells then were washed with PBS to remove nonadherent cells and detached by incubation in TrypLE Select at 37°C followed by gentle cell scraping. Macrophages were plated in 96-well plates at a density of 5 × 10^4^ cell/well and allowed to adhere overnight. Human macrophages were then polarized to an M1 activation state with 30 ng/mL human recombinant IFN-γ (R&D Systems, Bio-Techne, 285-IF-100) 100 ng/mL LPS (MilliporeSigma, L2630) for 24 hours before use.

### Macrophage phagocytosis.

Human or murine macrophages were pretreated for 15 minutes with RvD1 (1–100 nM) or vehicle control (0.1% ethanol) in respective growth media, which were then replaced with pHrodo Green *E*. *coli* BioParticles (Invitrogen, Thermo Fisher Scientific P35366) prepared in HBSS containing calcium and magnesium (HBSS^+/+^) and incubated at 37°C for 1 hour in the continued presence of RvD1 treatment. Unengulfed *E*. *coli* BioParticles were removed by washing with HBSS^+/+^, and intracellular fluorescence was measured using a 96-well plate reader at an excitation/emission of 509/533 nm. Experiments were performed in triplicate with cells derived from 3–4 donor hosts. Data are presented as the percentage change in fluorescence intensity when compared with vehicle-treated macrophages obtained from the same host.

### Data deposition.

The RNA-Seq data reported in this paper have been deposited in the Gene Expression Omnibus database (GSE146785).

### Statistics.

Data are presented as the mean ± SEM with raw data from each biological replicate shown. Statistical analysis was performed in GraphPad Prism 7. Between-group differences were tested by 2-tailed unpaired *t* tests (2 groups) or by a 1-way ANOVA followed by pairwise Holm-Šidák post hoc tests (≥3 groups). For time-course experiments, multiple-comparisons testing was done with a single baseline control group. *P* ≤ 0.05 was used to determine statistical significance.

### Study approval.

All animal experiments were approved by the University of Michigan Institutional Animal Care and Use committee (PRO00008744 and PRO00006079). Experiments with human participants were approved by the University of Michigan Institutional Review Board (HUM00158470).

## Author contributions

JFM and SVB conceived the study. SVB, KRM, PCDM, and CAA supervised the work. JFM, LAB, CAA, KBS, and SVB designed the experiments. JFM, LAB, EL, CF, JL, JACM, DCS, and CD performed the experiments. JFM, LAB, JL, and KRM analyzed the data. JFM prepared the figures and wrote the manuscript with input from all authors.

## Supplementary Material

Supplemental data

## Figures and Tables

**Table 1:**
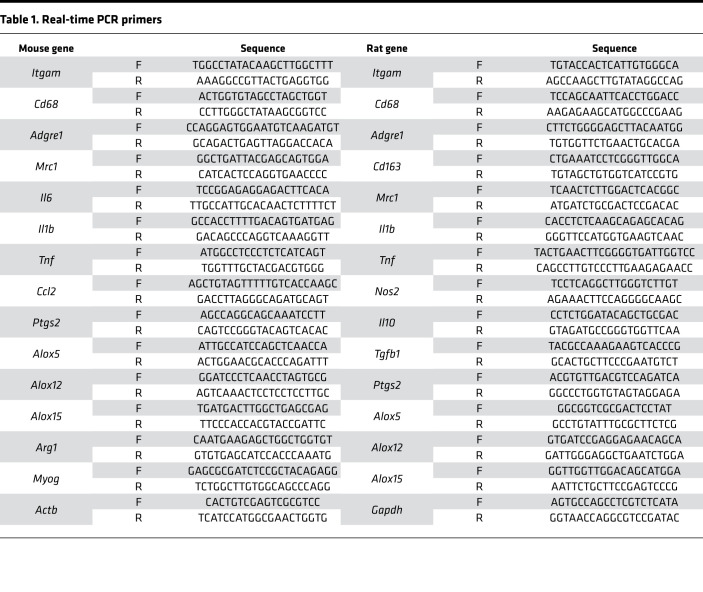
Real-time PCR primers

## Version 1 08/04/2020

In-Press Preview

## Version 2 09/17/2020

Electronic publication
