# Supplementary material for: Resolvin D1 supports skeletal myofiber regeneration via actions on myeloid and muscle stem cells
Source: JCI Insight. 2020 Sep 17;5(18):e137713. doi: 10.1172/jci.insight.137713 (PMC7526543; doi:10.1172/jci.insight.137713)
Supplement: Supplemental data [file jciinsight-5-137713-s033.pdf]

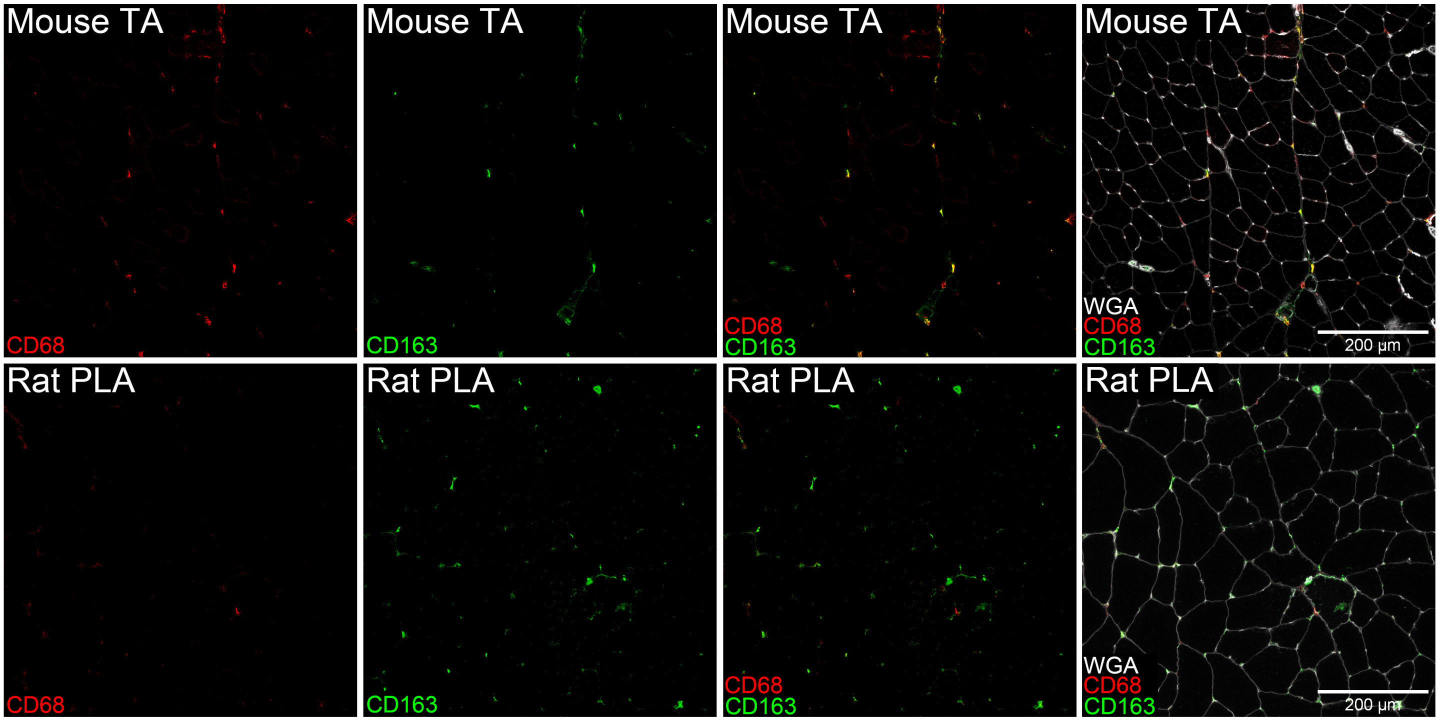

Supplemental Figure 1 – Resident skeletal muscle macrophage population: Cross-sections of uninjured mouse tibialis anterior (TA) and rat plantaris (PLA) muscles were stained with primary antibodies including rat anti-mouse CD68 (Bio-Rad, MCA1957, 1:50) or mouse anti-rat CD68 (ED1) (Abcam, ab31630, 1:50) in combination with rabbit polyclonal CD163 (ED2) (Santa Cruz, sc-33560, 1:50). Uninjured mouse TA muscles contained many resident CD163^{+} cells, the majority of which showed clear co-localization of CD68. In contrast, the many resident CD163^{+} (ED2) cells present in uninjured rat plantaris muscles showed little if any co-expression of the rat analog of CD68 (ED1).

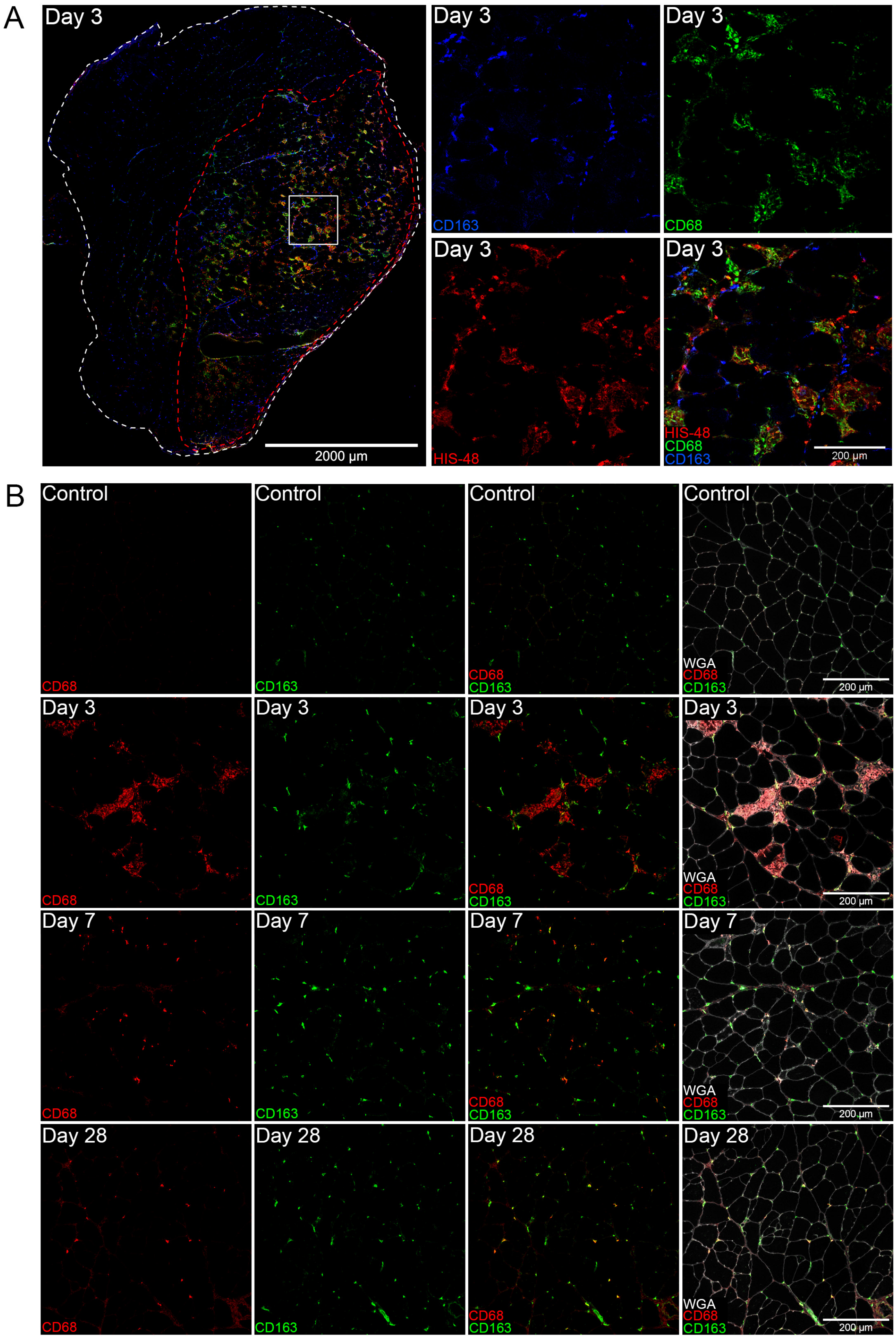

Supplemental Figure 2 – Functional overload induced skeletal muscle inflammation: A: Rat plantaris muscles at day 3 following functional overload induced by synergist ablation surgery were stained with a cocktail of antibodies for polymorphonuclear cells (PMNs, HIS48), ED1 macrophages (MΦ, CD68), and ED2 MΦ (CD163). At least three distinct innate immune cell types were present within the inflammatory zone (red outline) including PMNs (HIS48$^+$CD68$^-$CD163$^-$ cells), inflammatory ED1 monocyte/ MΦ (CD68$^+$CD163$^-$HIS48$^-$cells), and resident-like ED2 MΦ (CD163$^+$CD68$^-$HIS48$^-$cells). Many resident CD163$^+$ cells, but few CD68$^+$ or HIS48$^+$ cells were present outside of the inflammatory zone. B: At day 3 following synergist ablation surgery there was robust infiltration of the overloaded plantaris muscle by many inflammatory CD68$^+$CD163$^-$ cells (e.g. ED1 MΦ). At day 7 and 28 of synergist ablation smaller numbers of CD68$^+$ cells persisted within the overloaded plantaris, many of which showed a clear increase in the co-expression of CD163 (e.g. ED2 MΦ).

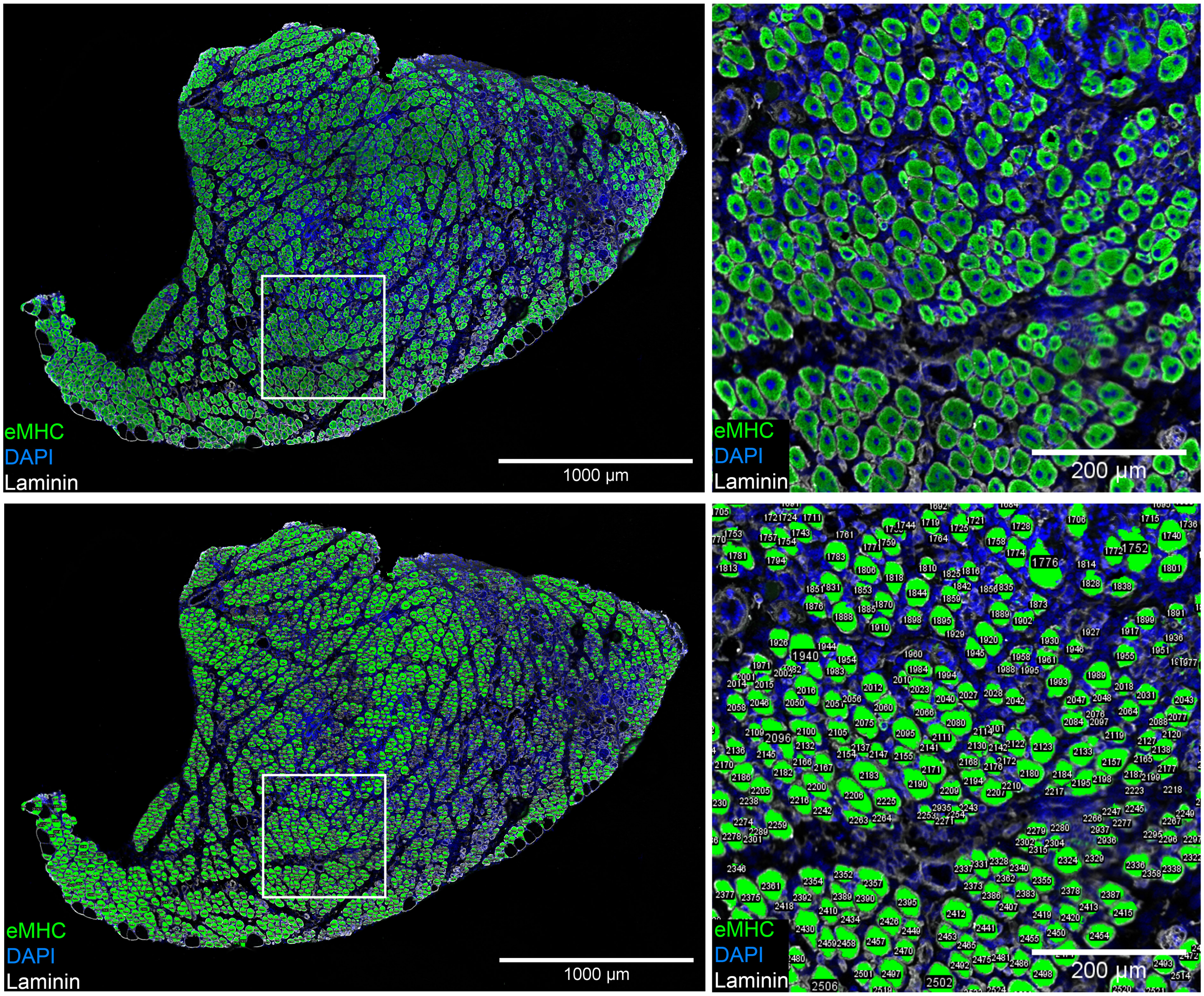

Supplemental Figure 3 – Representative staining and analysis of regenerating myofibers for embryonic myosin heavy chain (eMHC) following skeletal muscle injury: Mouse tibialis anterior (TA) muscles at day 5 following injury induced by intramuscular injection of barium chloride ($BaCl_2$) were stained with an antibody against eMHC (DSHB, F1.652s, 1:20). The number and size of each regenerating ($eMHC^+$) myofiber were analyzed on stitched panoramic images of the entire TA muscle section via semi-automated analysis using image J/FIJI software. Numeric labels mark those muscle fibers which were counted and measured by the software.

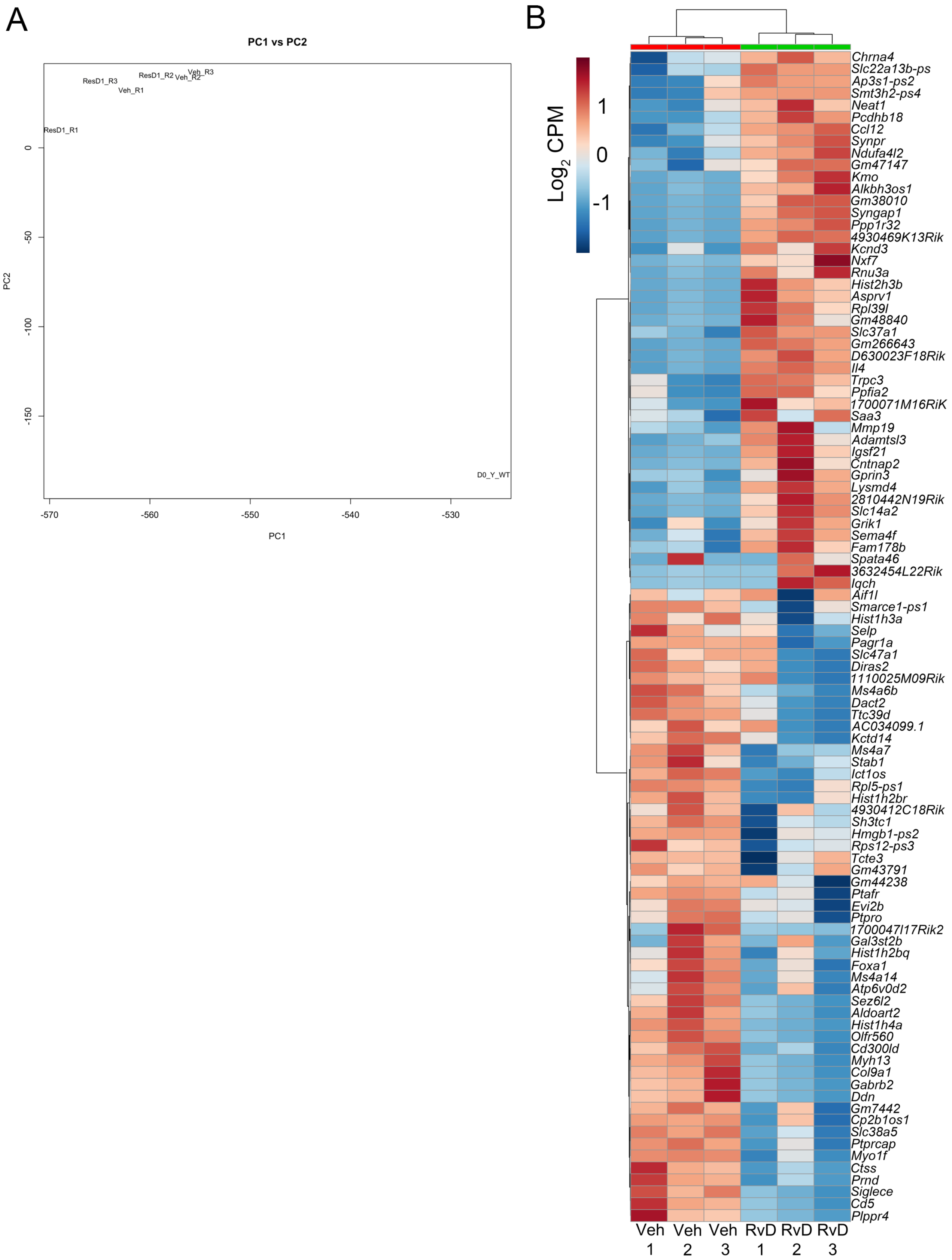

Supplemental Figure 4 – Muscle injury markedly impacts the global muscle satellite cell (MuSC) transcriptome with minimal overall effect of resolvin D1 (RvD1) treatment: A: Multidimensional scaling (MDS) plot showing results of unsupervised principle component analysis (PCA) of RNA sequencing (RNA-seq) data of the muscle satellite cell (MuSC) transcriptome. MuSCs were isolated from the tibialis anterior (TA) at day 3 following muscle injury induced by intramuscular injection of barium chloride ($BaCl_2$) for three biological replicates per group of mice receiving daily intraperitoneal injection with either resolvin D1 ("ResD1_R1-R3") or vehicle control ("Veh_R1-R3"). The transcriptome of MuSCs isolated from the entire hind-limb musculature of an age and gender matched uninjured control mouse is shown for comparison (D0_Y_WT). B: Top 100 muscle satellite cell (MuSC) genes modulated by resolvin D1 (RvD1) treatment following muscle injury. Heat map displaying the relative gene expression profiles of bulk MuSCs isolated from the tibialis anterior (TA) muscle as determined by RNA-sequencing at day 3 following injury induced by intramuscular injection of 50 µL of 1.2% barium chloride ($BaCl_2$) from six mice randomized to receive daily systemic treatment with either RvD1 (100 ng/mouse) or vehicle control (0.1% ethanol).

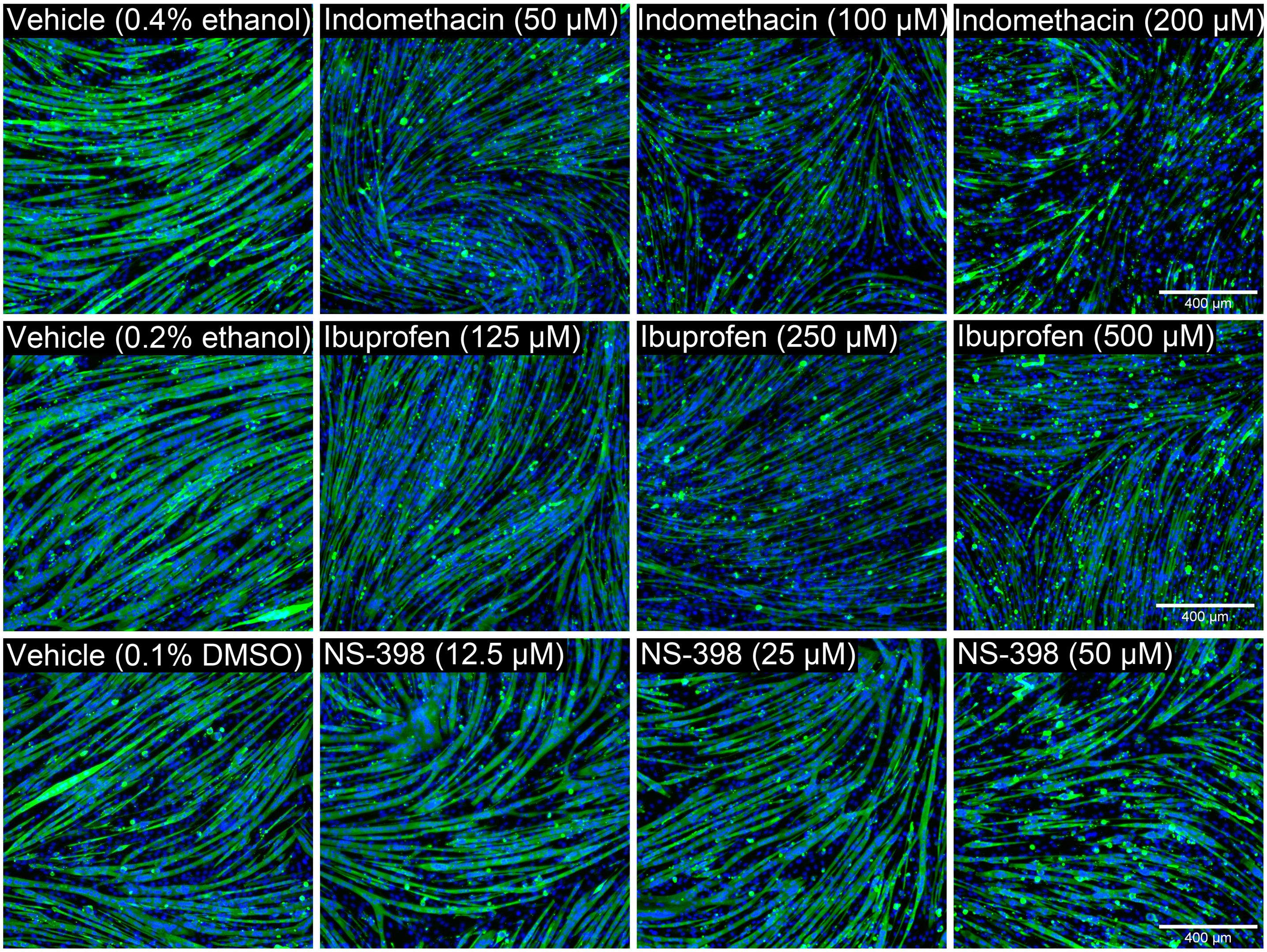

Supplemental Figure 5 – Dose-dependent inhibition of *in-vitro* myogenesis by non-steroidal anti-inflammatory drugs (NSAIDs): Myogenic precursor cells (C2C12 myoblasts) were induced to undergo myogenic differentiation via serum deprivation in the presence of increasing doses of NSAIDs including indomethacin, ibuprofen, and NS-398. At 3-days post-differentiation, the resulting fused myotube cultures were fixed in 4% paraformaldehyde (PFA) and stained with an antibody against sarcromeric myosin (DSHB, MF-20, 1:20). Cell nuclei were counterstained with DAPI and cells were visualized by fluorescence microscopy.

Supplemental Tables 1A: Cyclooxygenase metabolite concentration (pg/mg) in the mouse tibialis anterior (TA) muscle following $BaCl_2$ injury

Pathway	Substrate	Analyte	Sham 1D	Sham 3D	Sham 5D	$BaCl_2$ 1D	$BaCl_2$ 3D	$BaCl_2$ 5D
Cyclooxygenase	20:3n-6	PGE1	ND	ND	ND	ND	ND	2.49 ± 1.10
		PGF1alpha	ND	ND	ND	ND	ND	ND
		15-keto PGE1	ND	ND	ND	ND	ND	ND
		13,14dhPGE1	ND	ND	ND	ND	ND	ND
		13,14dh-15k-PGE1	ND	ND	ND	ND	ND	ND
		D17-PGE1	ND	ND	ND	ND	ND	ND
		15(R)-PGE1	ND	ND	ND	ND	ND	ND
		Bicyclo PGE1	ND	ND	ND	ND	ND	ND
		19(R)-hydroxy PGE1	ND	ND	ND	ND	ND	ND
		2,3-dinor PGE1	ND	ND	ND	ND	ND	ND
		6-keto PGE1	ND	ND	ND	ND	ND	ND
	20:4n-6	TXB2	9.08 ± 1.53	8.33 ± 1.03	7.09 ± 0.83	5.10 ± 0.32	27.14 ± 10.53	13.60 ± 3.89
		12-HHTrE	2.95 ± 0.34	2.47 ± 0.21	3.20 ± 0.30	2.15 ± 0.31	6.64 ± 2.15	7.00 ± 2.13
		11dh-2,3-dinor TXB2	2.52 ± 0.26	3.67 ± 0.48	5.48 ± 0.97	3.07 ± 0.27	3.56 ± 0.78	0.79 ± 0.48
		PGA2	4.66 ± 0.87	5.40 ± 1.53	4.52 ± 0.62	4.10 ± 0.41	7.47 ± 2.42	7.00 ± 1.23
		PGD2	10.61 ± 4.86	27.78 ± 15.50	5.40 ± 0.66	7.46 ± 3.45	60.63 ± 32.01	30.41 ± 11.60
		PGE2	31.91 ± 11.47	53.38 ± 17.51	27.63 ± 3.62	32.56 ± 8.46	69.11 ± 25.47	56.78 ± 13.64
		PGF2alpha	3.93 ± 0.32	4.73 ± 0.78	4.87 ± 0.22	3.20 ± 1.07	5.72 ± 0.98	7.67 ± 1.68
		6kPGF1alpha	3.55 ± 0.49	4.59 ± 0.84	4.51 ± 1.54	4.26 ± 0.82	10.43 ± 4.93	16.45 ± 5.06
		15-keto PGE2	0.68 ± 0.28	1.24 ± 0.33	1.01 ± 0.34	1.29 ± 0.09	2.45 ± 0.79	2.29 ± 0.88
		15-keto PGF2alpha	1.75 ± 0.75	3.30 ± 1.04	1.72 ± 0.17	2.04 ± 0.42	3.82 ± 1.29	3.64 ± 0.91
		13,14dh-15k-PGE2	2.08 ± 0.96	3.50 ± 1.46	2.01 ± 0.38	2.09 ± 0.64	4.74 ± 1.76	3.92 ± 1.09
		tetranor PGEM	ND	ND	ND	ND	ND	0.22 ± 0.12
		PGJ2	1.62 ± 0.30	2.74 ± 0.64	1.63 ± 0.69	1.44 ± 0.37	4.39 ± 1.75	3.56 ± 0.94
		D12-PGJ2	0.98 ± 0.52	1.58 ± 0.68	0.28 ± 0.26	0.76 ± 0.46	1.78 ± 0.93	1.21 ± 0.74
		13,14dh-15k-PGD2	1.75 ± 0.61	3.27 ± 0.92	1.63 ± 0.63	1.56 ± 0.66	4.69 ± 2.51	4.24 ± 1.64
		11dh-TXB2	ND	ND	ND	ND	ND	ND
		2,3-dinor TXB2	ND	ND	ND	ND	ND	ND
		13,14dh-15k-PGF2alpha	ND	ND	ND	ND	ND	ND
		8-isoPGF2alpha & 11bPGF2alpha	ND	ND	ND	ND	ND	ND
		19(R)-OH PGF2alpha & 20-OH PGF2alpha	ND	ND	ND	ND	ND	ND
		Bicyclo PGE2	ND	ND	ND	ND	ND	ND
		19(R)-OH PGE2 & 20-OH PGE2	ND	ND	ND	ND	ND	ND
		15d-D12,14-PGJ2	ND	ND	ND	ND	ND	ND
		6,15-diketo PGFalpha	ND	ND	ND	ND	ND	ND
		iPF-VI	ND	ND	ND	ND	ND	ND
	20:5n-3	TXB3	ND	ND	ND	ND	2.28 ± 0.93	ND
		11dh TXB3	ND	ND	ND	ND	ND	ND
		PGD3	ND	ND	ND	ND	ND	ND
		PGE3	ND	ND	ND	ND	ND	ND
		PGF3alpha	ND	ND	ND	ND	ND	ND
		15d-D12,14-PGJ3	ND	ND	ND	ND	ND	ND

Values are mean ± SEM of 5 mice/group. ND = Below limits of detection of the assay.

Supplemental Tables 1B: Lipoxygenase metabolite concentration (pg/mg) in the mouse tibialis anterior (TA) muscle following $BaCl_2$ injury

Pathway	Substrate	Analyte	Sham			$BaCl_2$		
			1D	3D	5D	1D	3D	5D
Lipoxygenase	18:2n-6	9-HODE	17.05 ± 3.04	18.93 ± 2.70	23.32 ± 2.05	19.79 ± 1.28	47.84 ± 12.90	47.88 ± 8.53
		9-OxoODE	21.28 ± 3.65	23.81 ± 4.40	27.24 ± 1.40	25.28 ± 2.09	54.09 ± 14.09	45.54 ± 15.21
		13-HODE	124.43 ± 19.14	129.16 ± 15.00	157.85 ± 17.91	124.45 ± 6.83	381.38 ± 111.39	312.06 ± 82.64
		13-OxoODE	37.07 ± 5.09	22.43 ± 3.71	54.17 ± 2.83	46.29 ± 6.46	98.27 ± 22.54	101.00 ± 32.05
	18:3n-3	9-HOTrE	1.09 ± 0.10	1.12 ± 0.34	1.83 ± 0.20	1.23 ± 0.18	2.76 ± 0.66	2.84 ± 0.93
		9-OxoOTrE	0.80 ± 0.05	0.95 ± 0.17	1.35 ± 0.09	0.80 ± 0.09	2.05 ± 0.60	2.28 ± 0.78
		13-HOTrE	2.19 ± 0.61	2.03 ± 0.12	3.15 ± 0.83	1.51 ± 0.20	8.98 ± 3.09	7.94 ± 3.60
	18:3n-6	13-HOTrE(gamma)	ND	ND	ND	ND	ND	ND
	20:2n-6	11-HEDE	0.22 ± 0.14	0.23 ± 0.15	0.17 ± 0.16	0.25 ± 0.14	0.51 ± 0.39	0.13 ± 0.12
		15-HEDE	0.15 ± 0.09	0.18 ± 0.07	0.17 ± 0.09	0.20 ± 0.08	1.14 ± 0.45	0.68 ± 0.33
		15-OxoEDE	ND	ND	ND	ND	ND	ND
	20:3n-9	5-HETrE	0.10 ± 0.03	0.17 ± 0.02	0.27 ± 0.11	0.12 ± 0.01	0.30 ± 0.05	0.44 ± 0.12
		8-HETrE	0.86 ± 0.20	0.95 ± 0.14	0.84 ± 0.20	0.88 ± 0.07	2.01 ± 0.55	1.93 ± 0.42
	20:4n-6	5-HETE	5.70 ± 1.45	5.73 ± 0.89	7.91 ± 2.22	4.05 ± 0.13	13.23 ± 3.74	25.38 ± 7.64
		5-oxoETE	1.14 ± 0.23	1.64 ± 0.10	1.43 ± 0.09	1.95 ± 0.17	3.39 ± 0.73	4.15 ± 1.26
		8-HETE	3.01 ± 0.78	2.31 ± 0.32	2.52 ± 0.43	2.49 ± 0.29	6.49 ± 1.72	7.55 ± 1.64
		9-HETE	ND	ND	ND	ND	ND	ND
		11-HETE	12.37 ± 1.67	14.45 ± 2.10	10.88 ± 0.53	12.99 ± 1.39	39.39 ± 12.54	41.83 ± 9.49
		12-HETE	338.65 ± 89.86	230.63 ± 55.65	124.93 ± 4.48	177.23 ± 30.88	930.19 ± 324.22	645.96 ± 278.41
		12-OxoETE	0.98 ± 0.46	0.38 ± 0.17	0.58 ± 0.22	0.78 ± 0.27	2.91 ± 1.15	1.55 ± 0.97
		tetranor 12-HETE	0.24 ± 0.11	0.44 ± 0.16	0.34 ± 0.17	0.05 ± 0.04	0.40 ± 0.16	0.45 ± 0.08
		15-HETE	7.71 ± 2.13	7.35 ± 0.67	7.54 ± 0.54	7.21 ± 0.87	24.77 ± 7.71	22.17 ± 5.93
		15-OxoETE	ND	ND	ND	ND	ND	ND
		20-HETE	4.03 ± 0.65	4.11 ± 0.81	3.11 ± 0.35	3.50 ± 1.00	8.86 ± 1.87	7.13 ± 1.61
		5(S),12(S)-DiHETE	11.86 ± 4.64	4.17 ± 0.71	3.22 ± 0.26	3.66 ± 1.08	25.92 ± 11.56	24.75 ± 13.75
		5(S),15(S)-DiHETE	ND	ND	ND	ND	ND	0.43 ± 0.31
		8(S),15(S)-DiHETE	ND	ND	ND	ND	ND	ND
		LTB4	ND	ND	ND	ND	ND	ND
		5(S),6(S)-DiHETE	ND	ND	ND	ND	ND	ND
		5,6-DiHETE(n-3))	ND	ND	ND	ND	ND	ND
		12-OxoLTB4	ND	ND	ND	ND	ND	ND
		20-hydroxy LTB4	ND	ND	ND	ND	ND	ND
		20-COOH LTB4	ND	ND	ND	ND	ND	ND
		18-carboxy dinor LTB4	ND	ND	ND	ND	ND	ND
	20:5n-3	5-HEPE	1.07 ± 0.23	1.24 ± 0.23	1.32 ± 0.15	0.83 ± 0.15	2.46 ± 0.78	3.16 ± 0.84
		8-HEPE	ND ± ND	ND ± ND	ND ± ND	ND ± ND	0.64 ± 0.39	0.83 ± 0.51
		9-HEPE	1.85 ± 0.92	0.78 ± 0.48	0.35 ± 0.33	0.92 ± 0.42	7.93 ± 3.14	5.30 ± 2.84
		11-HEPE	0.60 ± 0.30	0.55 ± 0.28	0.21 ± 0.19	0.56 ± 0.24	2.18 ± 0.70	2.59 ± 0.52
		12-HEPE	90.52 ± 17.66	59.47 ± 8.47	38.34 ± 3.72	36.66 ± 6.68	241.63 ± 80.93	165.77 ± 75.28
		15-HEPE	2.93 ± 1.49	2.50 ± 0.39	2.86 ± 1.24	2.03 ± 0.55	12.23 ± 5.19	7.73 ± 4.29
		18-HEPE	0.68 ± 0.32	0.80 ± 0.24	1.31 ± 0.12	0.95 ± 0.08	1.78 ± 0.37	2.33 ± 0.39
		LTB5	ND	ND	ND	ND	0.47 ± 0.28	ND
	22:6n-3	4-HDoHE	4.52 ± 0.64	4.82 ± 0.65	4.32 ± 0.30	4.82 ± 0.58	8.63 ± 1.86	9.18 ± 1.79
		7-HDoHE	1.15 ± 0.11	1.22 ± 0.11	1.08 ± 0.09	1.04 ± 0.32	2.37 ± 0.59	2.90 ± 0.29
		8-HDoHE	2.59 ± 0.29	2.34 ± 0.30	2.14 ± 0.20	2.50 ± 0.44	5.51 ± 1.46	4.70 ± 0.64
		10-HDoHE	5.21 ± 0.99	4.41 ± 0.22	3.55 ± 0.41	5.47 ± 0.69	12.98 ± 4.26	9.72 ± 2.45
		11-HDoHE	3.14 ± 0.99	2.71 ± 0.65	2.20 ± 0.25	2.65 ± 0.39	2.04 ± 1.06	3.89 ± 1.24
		13-HDoHE	3.76 ± 0.47	4.10 ± 0.41	3.59 ± 0.26	5.18 ± 0.85	11.39 ± 3.71	9.47 ± 1.55
		14-HDoHE	48.10 ± 10.93	31.80 ± 7.08	19.39 ± 3.50	36.35 ± 6.49	157.17 ± 65.68	76.54 ± 37.15
		16-HDoHE	4.19 ± 0.48	4.12 ± 0.61	4.40 ± 0.58	4.42 ± 0.46	8.82 ± 1.99	9.47 ± 1.10
		17-HDoHE	3.85 ± 1.66	3.35 ± 0.36	2.44 ± 0.86	3.79 ± 1.13	16.25 ± 7.07	8.61 ± 4.10
		20-HDoHE	2.77 ± 0.74	1.81 ± 1.10	3.88 ± 0.31	4.45 ± 0.61	6.81 ± 1.70	7.19 ± 1.19

Values are mean ± SEM of 5 mice/group. ND = Below limits of detection of the assay.

Supplemental Tables 1C: Epoxygenase metabolite concentration (pg/mg) in the mouse tibialis anterior (TA) muscle following $BaCl_2$ injury

Pathway	Substrate	Analyte	Sham 1D	Sham 3D	Sham 5D	$BaCl_2$ 1D	$BaCl_2$ 3D	$BaCl_2$ 5D
Epoxygenase	18:2n-6	9(10)-EpOME	311.33 ± 22.27	364.51 ± 65.00	409.82 ± 54.21	274.69 ± 64.10	675.21 ± 193.81	454.83 ± 87.73
		12(13)-EpOME	225.24 ± 20.36	253.95 ± 32.03	312.30 ± 37.50	227.99 ± 51.89	411.64 ± 102.42	275.60 ± 45.32
		9,10-DiHOME	10.47 ± 1.00	10.58 ± 0.87	15.98 ± 2.27	12.70 ± 1.49	16.29 ± 2.32	17.55 ± 2.65
		12,13-DiHOME	12.16 ± 1.35	11.08 ± 0.97	17.86 ± 2.50	15.49 ± 2.05	19.33 ± 2.90	19.14 ± 2.32
	20:4n-6	5(6)-EpETrE	1.53 ± 0.29	1.50 ± 0.18	1.21 ± 0.23	1.33 ± 0.15	2.63 ± 0.42	3.06 ± 0.81
		8(9)-EpETrE	ND	ND	ND	ND	4.66 ± 1.14	2.75 ± 2.12
		11(12)-EpETrE	17.34 ± 1.38	18.92 ± 2.64	16.60 ± 1.80	15.45 ± 2.06	36.47 ± 6.19	33.05 ± 7.36
		14(15)-EpETrE	8.91 ± 0.84	9.96 ± 1.34	9.52 ± 1.04	7.88 ± 1.08	19.59 ± 3.12	16.32 ± 3.18
		5,6-DiHETrE	ND	ND	ND	ND	ND	ND
		8,9-DiHETrE	ND	ND	ND	ND	ND	ND
		11,12-DiHETrE	0.71 ± 0.31	1.15 ± 0.17	1.56 ± 0.17	1.38 ± 0.12	2.28 ± 0.44	1.74 ± 0.48
		14,15-DiHETrE	1.69 ± 0.10	1.84 ± 0.19	2.73 ± 0.41	1.99 ± 0.19	3.55 ± 0.47	2.89 ± 0.19
	20:5n-3	8(9)-EpETE	ND	ND	ND	ND	ND	ND
		11(12)-EpETE	ND	ND	ND	ND	ND	ND
		14(15)-EpETE	ND	ND	ND	ND	ND	1.17 ± 0.74
		17(18)-EpETE	1.95 ± 0.21	1.58 ± 0.45	2.01 ± 0.80	1.16 ± 0.29	3.32 ± 1.03	3.17 ± 0.92
	22:6n-3	7(8)-EpDPE	3.75 ± 0.21	4.68 ± 1.01	2.95 ± 0.31	3.08 ± 0.44	7.27 ± 1.08	5.97 ± 1.11
		10(11)-EpDPE	23.55 ± 1.68	21.63 ± 3.28	20.83 ± 1.95	18.67 ± 3.50	41.73 ± 4.44	29.16 ± 4.90
		13(14)-EpDPE	12.58 ± 0.65	12.99 ± 1.96	11.53 ± 1.14	11.29 ± 1.68	22.63 ± 3.21	16.47 ± 2.26
		16(17)-EpDPE	9.18 ± 0.39	9.51 ± 1.51	8.13 ± 0.80	7.83 ± 1.40	15.11 ± 1.66	10.46 ± 1.77
		19(20)-EpDPE	3.95 ± 2.44	3.27 ± 3.25	6.04 ± 3.79	10.77 ± 3.56	24.31 ± 4.34	15.72 ± 3.18
		19,20-DiHDoPE	3.07 ± 0.91	2.46 ± 0.22	5.13 ± 2.17	11.79 ± 5.02	14.85 ± 5.69	6.42 ± 1.39

Values are mean ± SEM of 5 mice/group. ND = Below limits of detection of the assay.

Supplemental Tables 1D: Specialized pro-resolving mediators (pg/mg) in the mouse tibialis anterior (TA) muscle following $BaCl_2$ injury

Pathway	Substrate	Analyte	Sham 1D	Sham 3D	Sham 5D	$BaCl_2$ 1D	$BaCl_2$ 3D	$BaCl_2$ 5D
Specialized pro-resolving mediators	20:4n-6	LXA4	ND	ND	ND	ND	ND	ND
		LXB4	0.50 ± 0.30	0.85 ± 0.54	0.31 ± 0.29	ND	ND	0.71 ± 0.43
		15-epi LXA4	ND	ND	ND	ND	ND	ND
		15-oxo LXA4	ND	ND	ND	ND	ND	ND
	20:5n-3	LXA5	ND	ND	ND	ND	ND	ND
		RvE1	ND	ND	ND	ND	ND	ND
		RvE3	ND	ND	ND	ND	ND	ND
	22:6n-3	RvD1 & AT-RvD1	ND	ND	ND	ND	ND	ND
		RvD2	ND	ND	ND	ND	ND	ND
		RvD3	ND	ND	ND	ND	ND	ND
		AT-RvD3	ND	ND	ND	ND	ND	ND
		RvD4	ND	ND	ND	ND	ND	ND
		RvD5	ND	ND	ND	ND	ND	ND
		RvD6	ND	ND	ND	ND	ND	ND
		8-oxoRvD1	ND	ND	ND	ND	ND	ND
		17-oxoRvD1	ND	ND	ND	ND	ND	ND
		PD1	1.05 ± 0.68	3.88 ± 1.21	ND	3.74 ± 1.22	16.79 ± 6.70	ND
		AT-PD1	ND	ND	ND	ND	ND	ND
		10S,17S-DiHDoHE	ND	ND	ND	ND	ND	ND
		22-OH-PD1	ND	ND	ND	ND	ND	ND
		Maresin1	ND	ND	ND	ND	7.48 ± 3.67	ND
		7(S)-Maresin1	ND	ND	ND	ND	ND	ND
	22:5n-3	RvD5(n-3DPA)	ND	ND	ND	ND	ND	ND

Values are mean ± SEM of 5 mice/group. ND = Below limits of detection of the assay.

Supplemental Tables 1E: Cyclooxygenase metabolite concentration (pg/mg) in the rat plantaris muscle in response to functional overload

Pathway	Substrate	Analyte	Synergist ablation time-point: Control	3D	7D	28D
Cyclooxygenase	20:3n-6	PGE1	ND	ND	ND	ND
		PGF1alpha	ND	ND	ND	ND
		15-keto PGE1	ND	ND	ND	ND
		13,14dhPGE1	ND	ND	ND	ND
		13,14dh-15k-PGE1	ND	ND	ND	ND
		D17-PGE1	ND	ND	ND	ND
		15(R)-PGE1	ND	ND	ND	ND
		Bicyclo PGE1	ND	ND	ND	ND
		19(R)-hydroxy PGE1	ND	ND	ND	ND
		2,3-dinor PGE1	0.24 ± 0.12	0.08 ± 0.04	ND	ND
		6-keto PGE1	ND	ND	ND	ND
	20:4n-6	TXB2	5.54 ± 1.47	17.83 ± 5.76	5.21 ± 0.92	7.83 ± 2.67
		12-HHTrE	0.38 ± 0.11	1.45 ± 0.50	0.27 ± 0.10	0.69 ± 0.14
		11dh-2,3-dinor TXB2	1.83 ± 0.19	1.38 ± 0.13	1.75 ± 0.09	1.64 ± 0.17
		PGA2	1.52 ± 0.48	0.87 ± 0.33	0.86 ± 0.60	1.03 ± 0.42
		PGD2	1.79 ± 0.53	3.99 ± 1.89	3.04 ± 0.97	2.95 ± 0.85
		PGE2	2.66 ± 0.52	6.52 ± 1.23	4.57 ± 0.58	4.71 ± 0.93
		PGF2alpha	ND	0.46 ± 0.23	0.24 ± 0.16	0.22 ± 0.11
		6kPGF1alpha	ND	1.64 ± 0.18	0.69 ± 0.20	0.36 ± 0.18
		15-keto PGE2	0.59 ± 0.14	1.31 ± 0.25	1.24 ± 0.32	0.81 ± 0.21
		15-keto PGF2alpha	0.08 ± 0.03	0.18 ± 0.11	0.22 ± 0.11	0.14 ± 0.08
		13,14dh-15k-PGE2	ND	0.34 ± 0.08	ND	0.19 ± 0.09
		tetranor PGEM	ND	ND	ND	ND
		PGJ2	0.71 ± 0.11	0.47 ± 0.14	0.70 ± 0.16	0.86 ± 0.21
		D12-PGJ2	ND	ND	ND	ND
		13,14dh-15k-PGD2	ND	0.36 ± 0.23	ND	ND
		11dh-TXB2	ND	ND	ND	ND
		2,3-dinor TXB2	ND	ND	ND	ND
		13,14dh-15k-PGF2alpha	ND	ND	ND	ND
		8-isoPGF2alpha & 11bPGF2alpha	ND	ND	ND	ND
		19(R)-OH PGF2alpha & 20-OH PGF2alpha	ND	ND	ND	ND
		Bicyclo PGE2	ND	ND	ND	ND
		19(R)-OH PGE2 & 20-OH PGE2	ND	ND	ND	ND
		15d-D12,14-PGJ2	ND	ND	ND	ND
		6,15-diketo PGFalpha	ND	ND	ND	ND
		iPF-VI	ND	ND	ND	ND
	20:5n-3	TXB3	ND	ND	ND	ND
		11dh TXB3	ND	ND	ND	ND
		PGD3	ND	ND	ND	ND
		PGE3	ND	ND	ND	ND
		PGF3alpha	ND	ND	ND	ND
		15d-D12,14-PGJ3	ND	ND	ND	ND

Values are mean ± SEM of 8-12 muscles from 4-6 rats per group. ND = Below limits of detection of the assay.

Supplemental Tables 1F: Lipoxygenase metabolite concentration (pg/mg) in the rat plantaris muscle in response to functional overload

Pathway	Substrate	Analyte	Synergist ablation time-point Control	3D	7D	28D
Lipoxygenase	18:2n-6	9-HODE	10.20 ± 2.55	12.76 ± 3.62	9.36 ± 4.22	10.68 ± 2.52
		9-OxoODE	25.57 ± 4.54	29.34 ± 4.40	18.02 ± 2.83	42.75 ± 8.98
		13-HODE	86.61 ± 13.69	118.75 ± 25.86	69.53 ± 12.43	132.81 ± 26.42
		13-OxoODE	54.09 ± 10.69	72.70 ± 12.03	43.31 ± 6.13	85.30 ± 15.46
	18:3n-3	9-HOTrE	0.15 ± 0.07	0.70 ± 0.28	0.59 ± 0.17	0.77 ± 0.40
		9-OxoOTrE	0.97 ± 0.22	0.71 ± 0.16	0.53 ± 0.14	1.04 ± 0.21
		13-HOTrE	1.00 ± 0.27	2.39 ± 1.54	0.51 ± 0.24	0.97 ± 0.30
	18:3n-6	13-HOTrE(gamma)	ND	ND	ND	ND
	20:2n-6	11-HEDE	0.12 ± 0.05	0.13 ± 0.06	0.09 ± 0.04	0.29 ± 0.08
		15-HEDE	ND	0.42 ± 0.25	0.07 ± 0.03	0.23 ± 0.07
		15-OxoEDE	ND	0.06 ± 0.03	ND	ND
	20:3n-9	5-HETrE	0.15 ± 0.02	0.17 ± 0.03	0.11 ± 0.02	0.14 ± 0.03
		8-HETrE	0.15 ± 0.06	0.32 ± 0.08	0.21 ± 0.05	0.25 ± 0.07
	20:4n-6	5-HETE	4.86 ± 0.74	9.41 ± 1.18	5.68 ± 1.31	8.90 ± 1.92
		5-oxoETE	1.54 ± 0.29	2.71 ± 0.54	2.29 ± 0.40	2.83 ± 0.59
		8-HETE	2.83 ± 0.42	7.23 ± 1.79	3.34 ± 0.44	6.37 ± 1.33
		9-HETE	0.56 ± 0.24	1.20 ± 0.52	0.99 ± 0.46	1.30 ± 0.55
		11-HETE	9.41 ± 1.46	26.44 ± 6.30	11.84 ± 2.19	22.76 ± 4.37
		12-HETE	27.72 ± 7.44	270.16 ± 163.86	28.40 ± 10.25	46.81 ± 17.86
		12-OxoETE	ND	ND	ND	ND
		tetranor 12-HETE	0.35 ± 0.08	1.19 ± 0.16	0.32 ± 0.06	0.33 ± 0.14
		15-HETE	7.48 ± 0.67	20.12 ± 6.17	8.67 ± 1.82	9.47 ± 1.44
		15-OxoETE	0.51 ± 0.13	0.66 ± 0.13	0.88 ± 0.19	0.46 ± 0.19
		20-HETE	12.98 ± 1.89	35.70 ± 4.76	24.76 ± 7.07	42.13 ± 7.07
		5(S),12(S)-DiHETE	0.45 ± 0.24	22.58 ± 13.56	0.96 ± 0.40	2.16 ± 0.68
		5(S),15(S)-DiHETE	ND	0.41 ± 0.27	ND	ND
		8(S),15(S)-DiHETE	ND	ND	ND	ND
		LTB4	ND	ND	ND	ND
		5(S),6(S)-DiHETE	0.23 ± 0.16	1.11 ± 0.73	0.14 ± 0.08	0.41 ± 0.31
		5,6-DiHETE(n-3))	ND	ND	ND	ND
		12-OxoLTB4	ND	ND	ND	ND
		20-hydroxy LTB4	ND	ND	ND	ND
		20-COOH LTB4	ND	ND	ND	ND
		18-carboxy dinor LTB4	ND	ND	ND	ND
	20:5n-3	5-HEPE	0.63 ± 0.13	0.90 ± 0.22	0.78 ± 0.10	0.97 ± 0.18
		8-HEPE	ND	ND	ND	ND
		9-HEPE	ND	ND	ND	ND
		11-HEPE	ND	0.52 ± 0.28	0.24 ± 0.15	ND
		12-HEPE	3.27 ± 1.01	18.94 ± 8.20	2.30 ± 0.88	3.17 ± 1.07
		15-HEPE	0.24 ± 0.12	3.18 ± 2.93	0.44 ± 0.28	0.55 ± 0.31
		18-HEPE	1.32 ± 0.31	1.25 ± 0.26	0.92 ± 0.16	1.62 ± 0.30
		LTB5	ND	ND	ND	ND
	22:6n-3	4-HDoHE	4.69 ± 0.80	6.79 ± 1.10	3.67 ± 0.38	4.82 ± 0.74
		7-HDoHE	0.65 ± 0.17	1.02 ± 0.18	0.73 ± 0.25	0.90 ± 0.18
		8-HDoHE	1.92 ± 0.35	2.15 ± 0.69	1.53 ± 0.35	3.20 ± 0.76
		10-HDoHE	1.92 ± 0.47	4.24 ± 0.88	1.84 ± 0.34	2.62 ± 0.59
		11-HDoHE	1.49 ± 0.33	5.04 ± 1.69	1.19 ± 0.44	2.61 ± 0.45
		13-HDoHE	2.01 ± 0.27	3.97 ± 0.96	1.93 ± 0.32	3.08 ± 0.59
		14-HDoHE	3.77 ± 1.06	11.50 ± 3.23	3.31 ± 0.89	4.68 ± 1.44
		16-HDoHE	3.43 ± 0.55	5.48 ± 0.81	3.56 ± 0.87	3.64 ± 0.35
		17-HDoHE	1.20 ± 0.33	4.88 ± 2.20	1.08 ± 0.26	0.93 ± 0.20
		20-HDoHE	2.61 ± 0.46	3.33 ± 0.53	2.62 ± 0.31	3.20 ± 0.46

Values are mean ± SEM of 8-12 muscles from 4-6 rats per group. ND = Below limits of detection of the assay.

Supplemental Tables 1G: Epoxygenase metabolite concentration (pg/mg) in the rat plantaris muscle in response to functional overload

Pathway	Substrate	Analyte	Synergist ablation time-point			
			Control	3D	7D	28D
Epoxygenase	18:2n-6	9(10)-EpOME	553.08 ± 166.37	966.86 ± 308.22	375.76 ± 88.30	1416.53 ± 413.19
		12(13)-EpOME	390.72 ± 133.24	552.96 ± 173.27	181.85 ± 46.76	901.26 ± 267.93
		9,10-DiHOME	7.95 ± 1.30	13.58 ± 3.45	10.30 ± 2.95	12.69 ± 3.57
		12,13-DiHOME	14.57 ± 2.78	18.83 ± 4.20	10.74 ± 2.14	19.81 ± 7.66
	20:4n-6	5(6)-EpETrE	14.89 ± 4.18	35.35 ± 10.04	8.40 ± 2.00	18.03 ± 3.42
		8(9)-EpETrE	16.96 ± 16.96	47.74 ± 47.74	21.56 ± 21.56	57.91 ± 57.91
		11(12)-EpETrE	83.72 ± 31.23	167.89 ± 44.61	83.37 ± 24.71	194.47 ± 36.23
		14(15)-EpETrE	37.58 ± 13.24	62.42 ± 17.85	38.79 ± 6.90	121.60 ± 28.26
		5,6-DiHETrE	ND	0.62 ± 0.50	ND	ND
		8,9-DiHETrE	ND	ND	ND	ND
		11,12-DiHETrE	0.99 ± 0.23	3.06 ± 1.11	0.50 ± 0.25	0.64 ± 0.19
		14,15-DiHETrE	1.60 ± 0.38	3.62 ± 0.92	2.21 ± 0.78	2.76 ± 1.22
	20:5n-3	8(9)-EpETE	ND	ND	0.85 ± 0.48	0.91 ± 0.59
		11(12)-EpETE	ND	ND	ND	ND
		14(15)-EpETE	1.77 ± 0.92	3.25 ± 1.39	2.81 ± 1.17	5.05 ± 3.87
		17(18)-EpETE	6.45 ± 2.33	5.78 ± 1.46	2.79 ± 0.81	9.30 ± 1.76
	22:6n-3	7(8)-EpDPE	8.65 ± 3.19	13.68 ± 3.42	6.95 ± 2.28	12.99 ± 3.88
		10(11)-EpDPE	37.57 ± 11.25	85.23 ± 21.60	30.40 ± 8.27	69.22 ± 13.60
		13(14)-EpDPE	21.68 ± 7.16	38.01 ± 16.76	15.46 ± 4.74	43.08 ± 10.02
		16(17)-EpDPE	14.56 ± 5.18	20.16 ± 5.66	9.32 ± 2.25	21.45 ± 3.89
		19(20)-EpDPE	16.87 ± 6.48	28.21 ± 8.89	9.73 ± 2.85	18.90 ± 5.59
		19,20-DiHDoPE	ND	ND	ND	ND

Values are mean ± SEM of 8-12 muscles from 4-6 rats per group. ND = Below limits of detection of the assay.

Supplemental Tables 1H: Specialized pro-resolving mediator concentration (pg/mg) in the rat plantaris muscle in response to functional overload

Pathway	Substrate	Analyte	Synergist ablation time-point			
			Control	3D	7D	28D
Specialized pro-resolving mediators	20:4n-6	LXA4	0.19 ± 0.11	0.70 ± 0.34	0.18 ± 0.11	1.07 ± 0.79
		LXB4	0.33 ± 0.21	0.20 ± 0.13	0.29 ± 0.21	0.42 ± 0.30
		15-epi LXA4	ND	ND	ND	0.68 ± 0.60
		15-oxo LXA4	ND	ND	ND	ND
	20:5n-3	LXA5	ND	ND	ND	ND
		RvE1	ND	ND	ND	ND
		RvE3	ND	ND	ND	ND
	22:6n-3	RvD1 & AT-RvD1	ND	ND	ND	ND
		RvD2	ND	ND	ND	ND
		RvD3	ND	ND	ND	ND
		AT-RvD3	ND	ND	ND	ND
		RvD4	ND	ND	ND	ND
		RvD5	ND	ND	ND	ND
		RvD6	ND	0.59 ± 0.28	ND	0.18 ± 0.11
		8-oxoRvD1	6.75 ± 2.63	13.15 ± 3.65	6.24 ± 1.87	9.07 ± 3.08
		17-oxoRvD1	ND	ND	ND	ND
		PD1	0.93 ± 0.53	28.78 ± 11.91	0.56 ± 0.54	2.31 ± 1.70
		AT-PD1	ND	ND	ND	ND
		10S,17S-DiHDoHE	ND	18.90 ± 12.93	ND	ND
		22-OH-PD1	ND	ND	ND	ND
		Maresin1	ND	9.72 ± 7.17	ND	ND
		7(S)-Maresin1	ND	ND	ND	ND
	22:5n-3	RvD5(n-3DPA)	ND	ND	ND	ND

Values are mean ± SEM of 8-12 muscles from 4-6 rats per group. ND = Below limits of detection of the assay.
